# Abundant RNA editing sites of chloroplast protein-coding genes in *Ginkgo biloba* and an evolutionary pattern analysis

**DOI:** 10.1186/s12870-016-0944-8

**Published:** 2016-12-01

**Authors:** Peng He, Sheng Huang, Guanghui Xiao, Yuzhou Zhang, Jianing Yu

**Affiliations:** College of life sciences, Shaanxi Normal University, Xi’an, China

**Keywords:** RNA editing, Posttranscriptional modification, *Ginkgo biloba*, Chloroplast genome, Protein structure

## Abstract

**Background:**

RNA editing is a posttranscriptional modification process that alters the RNA sequence so that it deviates from the genomic DNA sequence. RNA editing mainly occurs in chloroplasts and mitochondrial genomes, and the number of editing sites varies in terrestrial plants. Why and how RNA editing systems evolved remains a mystery. *Ginkgo biloba* is one of the oldest seed plants and has an important evolutionary position. Determining the patterns and distribution of RNA editing in the ancient plant provides insights into the evolutionary trend of RNA editing, and helping us to further understand their biological significance.

**Results:**

In this paper, we investigated 82 protein-coding genes in the chloroplast genome of *G. biloba* and identified 255 editing sites, which is the highest number of RNA editing events reported in a gymnosperm. All of the editing sites were C-to-U conversions, which mainly occurred in the second codon position, biased towards to the U_A context, and caused an increase in hydrophobic amino acids. RNA editing could change the secondary structures of 82 proteins, and create or eliminate a transmembrane region in five proteins as determined *in silico*. Finally, the evolutionary tendencies of RNA editing in different gene groups were estimated using the nonsynonymous-synonymous substitution rate selection mode.

**Conclusions:**

The *G. biloba* chloroplast genome possesses the highest number of RNA editing events reported so far in a seed plant. Most of the RNA editing sites can restore amino acid conservation, increase hydrophobicity, and even influence protein structures. Similar purifying selections constitute the dominant evolutionary force at the editing sites of essential genes, such as the *psa,* some *psb* and *pet* groups, and a positive selection occurred in the editing sites of nonessential genes, such as most *ndh* and a few *psb* genes.

**Electronic supplementary material:**

The online version of this article (doi:10.1186/s12870-016-0944-8) contains supplementary material, which is available to authorized users.

## Background

In the plastids and mitochondria of land plants, mature transcripts are profoundly affected by RNA editing, which alters the genetic information of the RNA molecules [1]. RNA editing was first documented in the *coxII* gene of a trypanosome. Comparisons of the *coxII* transcript with homologous genes of other species showed that the open reading frame of this gene in *Trypanosoma brucei* shifts due to the addition of a nucleotide in the transcript, resulting in a new readable frame [2]. In plants, RNA editing was found for the first time in the *coxII* of *Triticum aestivum* [3]. Two years later, the RNA editing of the *rpl2* transcript was reported in maize, which produced an initiation codon, ATG, derived from ACG [4]. To date, more than 200 higher plant chloroplast genomes have been sequenced, but editing sites were completely detected only in one moss (*Anthoceros formosae*) [5], one fern (*Adiantum capillus-veneris*) [6], two gymnosperm (*Pinus thunbergii* and *Cycas taitungensis*) [7, 8], seven eudicots (*Atropa belladonna*, *Solanum lycopersicum*, *Phalaenopsis aphrodite*, *Cucumis sativus*, *Arabidopsis thaliana, Nicotiana tabacum* and *Gossypium hirsutum*) [9–15], and four monocotyledons (*Oryza sativa*, *Saccharum officinarum*, *Triticum aestivum* and *Zea mays*) [16–18]*.*

In higher plants, RNA editing mainly occurs in the protein-encoding genes of mitochondria and chloroplasts and it mostly converts C to U, although hornwort and fern have abundant U to C editing. Moreover, the editing events have also been detected in tRNAs, introns and the untranslated regions [19, 20]. RNA editing is essential for the normal development of plant and is involved in a wide variety of biological pathways. For example, RNA editing has been associated with cytoplasmic male sterility [21, 22]. The rice *atp9* transcript of a cytoplasmic male sterile line has no editing sites, while the transcript of the maintainer line has two editing sites, which changes the amino acid sequence of the protein [23]. Cao et al. found editing efficiencies are significantly reduced at the *accD*-794, *accD*-1568 and *ndhF*-290 sites, which could lead to etiolating and the delayed greening phenotype at the young seeding stage in *A. thaliana* [24].

The evolutionary pattern of RNA editing is another interesting topic. Some scholars believe that the RNA editing phenomenon is a relic of ancient RNA world and is involved in primordial error correction, such as repairing UV damage at the transcript level. Others argue that the editing system produces additional mutations to adapt to different physiological functions. However, this does not explain why RNA editing did not occur in some ancient predating parasitic organisms [25]. Although one model, constructive neutral evolution, proposed that the RNA editing mechanism might randomly emerge and be suppressed in some primordial living organisms [26, 27]. How RNA editing systems evolved remains controversial.

*Ginkgo biloba L.* (Ginkgoaceae) is one of the oldest seed plants, a living fossil with evidence indicating it has existed on earth for 270 million years, and it occupies an important phylogenetic position in plant evolution [28–30]. The gene map of the *G. biloba* chloroplast genome was released in 2012 (Accession number: AB684440). The full-length chloroplast genome is 156,945 bp and contains 82 protein-coding genes, 35 tRNA genes and 4 rRNA genes [31]. Investigating the RNA editing sites in *G. biloba* may provide us with evolutionary insights on how RNA editing systems varied during the evolution of terrestrial plants and on which editing sites may be retained to execute functions.

In this paper, we explored the RNA editing sites of the protein-encoding genes in the *G. biloba* chloroplast genome, and identified 255 editing sites in 82 transcripts, which is the highest number of RNA editing cases reported in seed plants. Many of the editing sites in *G. biloba* are unique and are mainly distributed in the NADH-dehydrogenase complex (*ndh*) genes. In addition, bioinformatics analysis showed that RNA editing can restore amino acid conservation, increase hydrophobicity, and influence the proteins’ secondary or tertiary structure. Finally, the evolutionary tendencies of RNA editing in different gene groups were estimated using the nonsynonymous-synonymous substitution rate (dN-dS) selection mode, and the results showed that similar purifying and positive selections constituted the dominant evolutionary force at the RNA editing sites of essential and unessential genes, respectively.

## Methods

### Plant materials and growth conditions

*Ginkgo biloba L*. (Ginkgoaceae) seedlings were harvested from Xi’an botanical garden (E, 108°93′, N, 34°17′, Shaanxi Province, Northwest China) and grown in a greenhouse under long-day conditions (16-h light/8-h dark cycle) at 28 ± 2 °C. Leaves were harvested from 8-week-old plants, and frozen in liquid nitrogen.

### DNA isolation and PCR

The DNA was isolated using an improved CTAB protocol. Plant leaves (0.1 g) were ground into powder in liquid nitrogen. Then, 0.6 mL CTAB extraction buffer was added and the lysate was incubated at 65 °C for 30 min. The DNA was purified by adding an equal volume of a mixture of chloroform: isoamyl alcohol (24:1) followed by centrifugation at 8000 × *g* for 10 min at 4 °C. The supernatant was added to 2/3 volume of isopropanol and then subjected to centrifugation at 8000 × *g*. The precipitate was washed twice with 75% ethanol and then dissolved in 300 μL sterile water. NaAc (1/10 volume of 3 M, pH 5.2) and two volumes of ethanol were added to the tube followed by a 10-min incubation at −20 °C. The tube was centrifuged at 8000 × *g* for 5 min and the pellet was then washed twice with 75% ethanol and re-dissolved in 20 μL sterile water.

The primers of 82 *G. biloba* transcripts were designed based on the *G. biloba* chloroplast complete genome [AB684440], and the primer sequences are listed in (Additional file 1: Table S1). The PCRs were performed as follows: 95 °C for 3 min, 94 °C denaturing for 30 s, 53–60 °C annealing for 30 s, and an elongating time between 30 s and 1.5 min at 72 °C based on the DNA length (1 min per 1 kb). The PCR amplification products were electrophoresed on a 1% agarose gel and purified with E.Z.N.A^TM^ Gel Extraction Kit (OMEGA Bio-Tek, USA). The direct sequencing of cDNAs derived from these transcripts and of the corresponding genomic DNA (gDNA) was carried out by Sangon Biological Engineering Technology & Services (Shanghai, China).

### RNA isolation and RT-PCR

The total RNA was extracted using E.Z.N.A^TM^ Plant RNA Kit according to the manufacturer’s protocol. The tissue was disrupted and homogenized as above, and the gDNA was preliminarily eliminated with a gDNA filter. The flow-through at the very last step was mixed with the membrane-binding solution and then loaded into the HiBind RNA Mini column. Finally, RNA was washed with RWC buffer and RNA wash buffer to remove protein, polysaccharide and salt contamination. The total RNA was treated with DNaseI to remove gDNA contamination. The cDNA was synthesized according to the PrimeScript RT Reagent Kit protocol (TaKaRa, Dalian, China).

### RNA editing site identification

Direct sequencing was used in this paper. The PCR products were purified and sequenced at least three times. The editing sites were detected by aligning the DNA and cDNA sequences one by one using the EMBL-EBI ClustalW (http://www.ebi.ac.uk/Tools/msa/clustalo/). The sequences were analyzed using SeqMan of the Lasergene software package (https://www.dnastar.com/t-seqmanpro.aspx). According to Mower and Palmer [32], T and C appeared at the same site and clearly above the background, indicating partially edited sites.

### Analysis of the protein structures, and their composition before and after editing

MegAlign of the Lasergene package was used to analyze protein similarities. The N-terminal signal peptide prediction was carried out by SignalP (http://www.cbs.dtu.dk/services/SignalP), and SOPMA (https://npsa-prabi.ibcp.fr/cgi-bin/npsa_automat.pl?page=/NPSA/npsa_sopma.html) was employed to analyze the changes in the secondary structure. TMHMM (http://www.cbs.dtu.dk/services/TMHMM/) was used to predict alterations in the transmembrane region.

### Evolution analysis of RNA editing genes

For the RNA editing evolutionary analysis, the *ndh*, *pet*, *psa* and *psb* gene families from 12 species were selected, and then a z-test was applied to detect selection constraints using Mega 5.1 software. The non-synonymous–synonymous (dN–dS) substitution rate analysis was also conducted for each gene according to the Goldman and Yang (GY-94) method in Hyphy, which estimates dS and dN substitution rates through a codon-based model [33–35]. Parameters were set as follows: Test hypothesis mode was set as Neutrality. Nei-Gojobori method was chosen in the substitution mode. In general, a dN value lower than dS (dN < dS) suggests negative selection, i.e. nonsilent substitutions have been purged by natural selection, whereas the inverse scenario (dN > dS) implies positive selection, i.e. advantageous mutations have accumulated during the course of evolution.

### The homologues gene sequences and editing sites used in this paper

The 12 species used for the sequence alignments are listed as follows: *A. belladonna* [NC_004561.1]; *S. lycopersicum* [AM087200]; *C. sativus* [AJ970307]; *A. formosae* [NC_004543.1]; *G. hirsutum* [DQ345959.1]; *A. thaliana* [NC_000932.1]; *C. taitungensis* [NC_009618]; *A. capillus-veneris* [AY178864.1]; *T. aestivum* [AB042240.3]; *N. tabacum* [Z00044.2]; *Z. mays* [NC_001666.2]; *G. biloba* [AB684440.1]. Most of the editing site information was acquired from GenBank and RNA (http://dna.kdna.ucla.edu/rna/index.aspx) databases. Some editing sites were found in the literature.

## Results

### *G. biloba* chloroplast transcripts undergo several editing events

Based on the sequence alignments between DNAs and cDNAs, we identified 255 editing sites in 82 protein-coding genes in the *G. biloba* chloroplast genome, and all of the editing sites were C-to-U conversions. Among the 255 editing sites, *ycf3* (407 and 408 bp, nucleotide position in the gene’s coding sequence), *psbB* (1391 and 1392 bp), *rps14* (193 and 194 bp) and *ndhD* (1995 and 1996 bp) had two editing sites within one codon. RNA editing also created two new start codons in *petL* and *rps8*, and seven stop codons in *ccsA*, *rps4*, *rps18*, *petD*, *petL*, *ndhC* and *ndhK* (Additional file 2: Table S2). In addition, the highest number of partial editing sites was found in the transcripts of *G. biloba* compared with that in transcripts of other spermatophytes. A total of 73 partial editing sites occurred at the first (23), second (45) and third (5) codon positions. *ndhD* has the highest editing frequency, followed by *ndhA*, *ndhB*, *ndhK*, *rpoC1*, *matK* and *rpoA*. Additionally, *ndhF* has 18 partial editing sites, which is the highest number of partial editing sites in one gene. There are 16 silent editing sites, which cannot alter the corresponding amino acids, in 14 transcripts, *ycf1*, *ycf3*, *ycf4*, *psbA*, *psbC*, *psbD*, *ndhD, ndhF*, *ndhK*, *petA*, *rpl2*, *rpoA*, *rpoB* and *chlN* (Table 1).

**Table 1:** Silent editing sites in chloroplast genes of *Ginkgo biloba*

Gene	Codon position	Codon change	Amino acid change
*ycf1*	634	Cua → Uua	Leu → Leu
*ycf3*	30	uuC → uuU	Phe → Phe
*ycf4*	15	ucC → ucU	Ser → Ser
*psbA*	804	ucC → ucY	Ser → Ser
*psbC*	876	uuC → uuY	Phe → Phe
*psbD*	894	gaC → gaU	Asp → Asp
*ndhD*	594	cuC → cuU	Leu → Leu
*ndhF*	1242	uuC → uuY	Phe → Phe
*ndhK*	69	cuC → cuU	Leu → Leu
	81	ucC → ucU	Ser → Ser
*petA*	615	guC → guU	Val → Val
*rpl2*	282	cuC → cuU	Leu → Leu
*rpoA*	100	Cua → Uua	Leu → Leu
*rpoB*	3029	cuC → cuY	Leu → Leu
*chlN*	118	Cua → Yua	Leu → Leu
	151	Cua → Yua	Leu → Leu

**Fig. 1:**
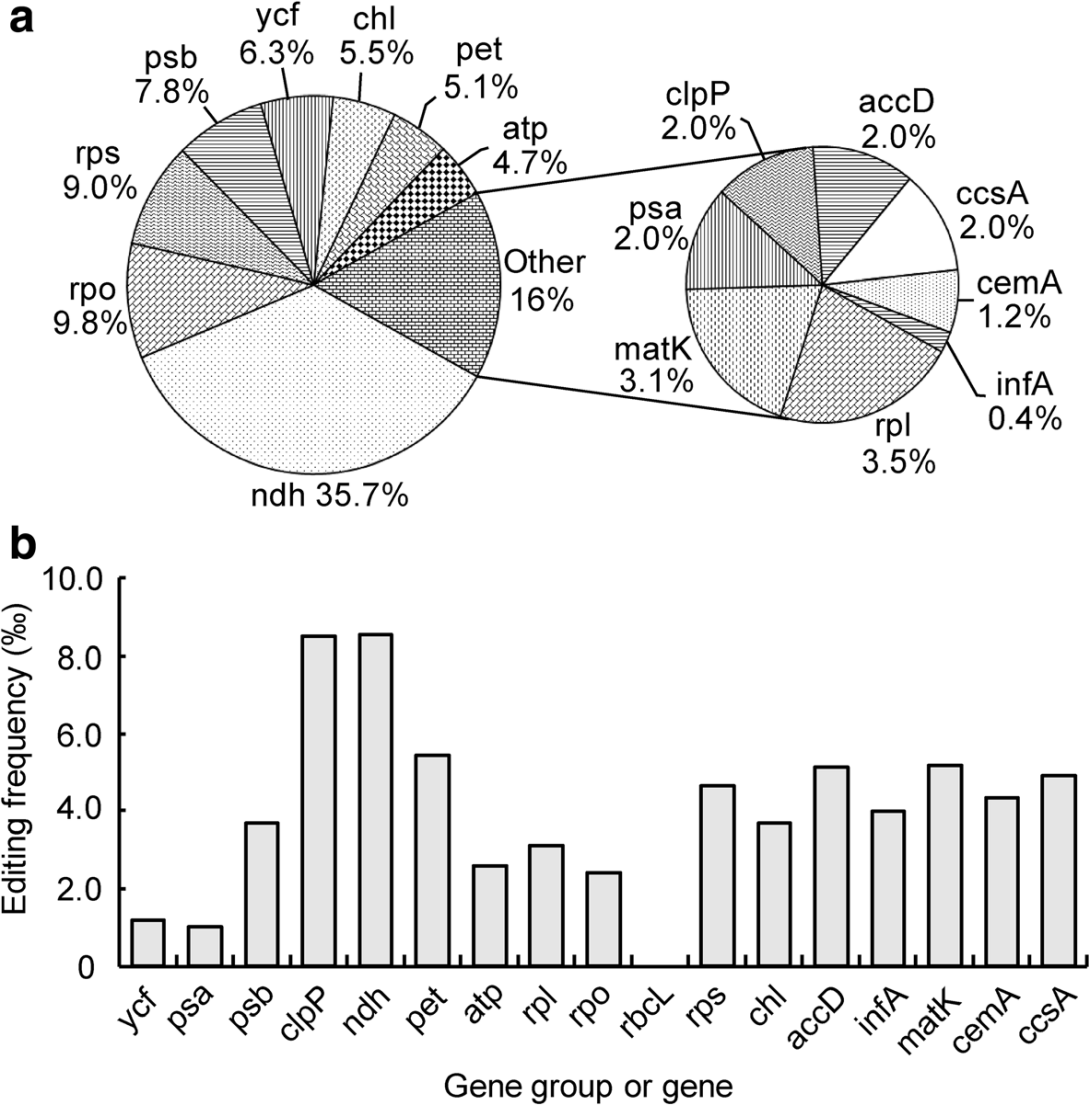
The distribution of editing sites and editing frequencies in the chloroplast genes of *Ginkgo biloba.* **a** The distribution of editing sites in the chloroplast genes of *Ginkgo biloba*. **b** The editing frequencies of *Ginkgo biloba* chloroplast genes. Editing frequency is indicated as the percentage of editing sites per analyzed base (bp)

We further analyzed the RNA editing frequencies of different gene groups in the chloroplast genome of *G. biloba*. The results showed that *ndh* genes exhibited the most editing cases, which were nearly 36% of the total editing sites, while the number of cases was not more than 10% in other genes (Fig. 1a).

To exclude interference by the gene length on the editing events, the number of corresponding editing sites was divided by the length of each gene group. *ndh* and *clpP* exhibited the same, and the highest, editing frequency, up to 8.5‰. Interestingly, *rbcL* had an almost undetectable editing frequency (Fig. 1b). These data suggested that *ndh* genes are more likely to be edited than other genes at the mRNA level.

### The characteristics of the RNA editing sites in the *G. biloba* chloroplast genome

**Fig. 2:**
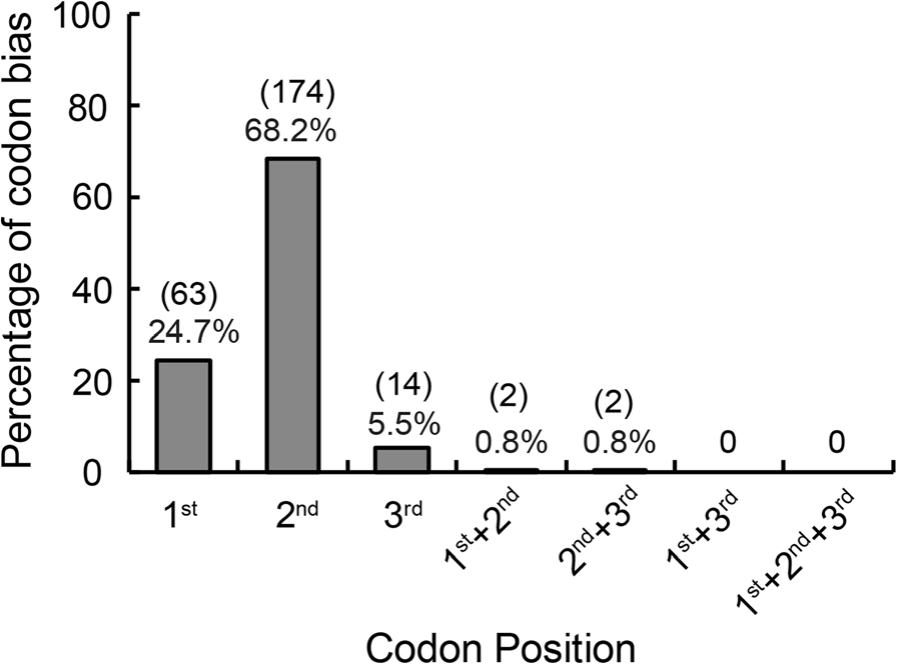
The codon bias at *Ginkgo biloba* chloroplast RNA editing sites. 1st, 2nd, 3rd indicates editing sites in the first, second, and third positions in the codon, respectively. 1st + 2nd, 2nd + 3rd, 1st + 3rd 1st + 2nd + 3rd indicate editing in first and second positions, second and third positions, first and third positions, and editing in the three codon positions, respectively. Percentage of codon bias shows the proportion of the positional preference. The numbers in the bracket are the number of editing events occurring at the position

**Fig. 3:**
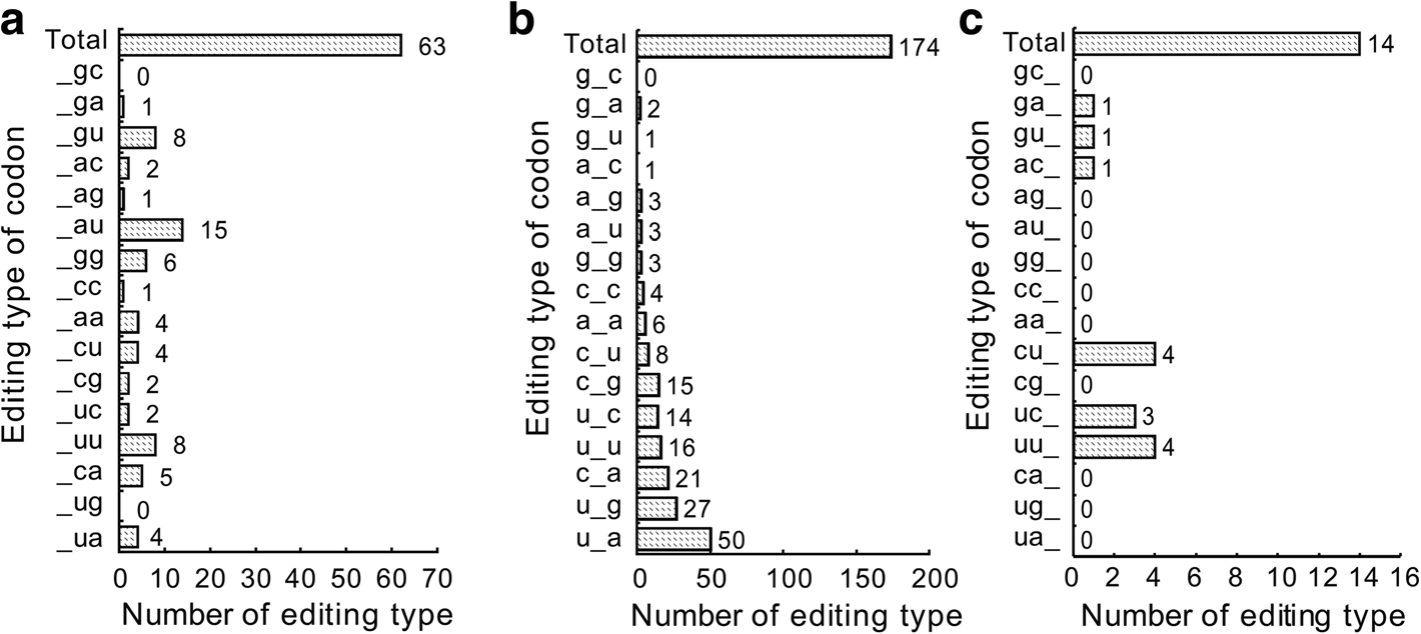
The RNA editing codon background of *Ginkgo biloba*. **a** C-U editing occurs at the first position of the codon. **b** C-U editing occurs at the second position of the codon. **c** C-U editing occurs at the third position of the codon

To gain further insights into the characteristics of the 255 RNA editing sites in the *G. biloba* chloroplast genome, we analyzed different types of editing codon positions. There were 63, 174 and 14 editing sites occurring at the first, second and third codon positions, respectively (Fig. 2). Editing sites occurred in second or third positions in one codon of the *ycf3, psbB, rps14* and *ndhD* transcripts (Fig. 2, Additional file 2: Table S2). For the editing sites distributed in the first codon positions, there are 37 sites in front of purine (adenine or guanine at the second codon position), which makes up ~59% of the editing occurring in the first codon positions. In the second codon position, editing occur in a U_A context (50), followed by U_G (27), C_A (21), U_U (16), C_G (15) and U_C (14) context (the numbers in parentheses refer to the number of RNA editing sites in which editing occurred at the second position in a codon) (Fig. 3).

**Fig. 4:**
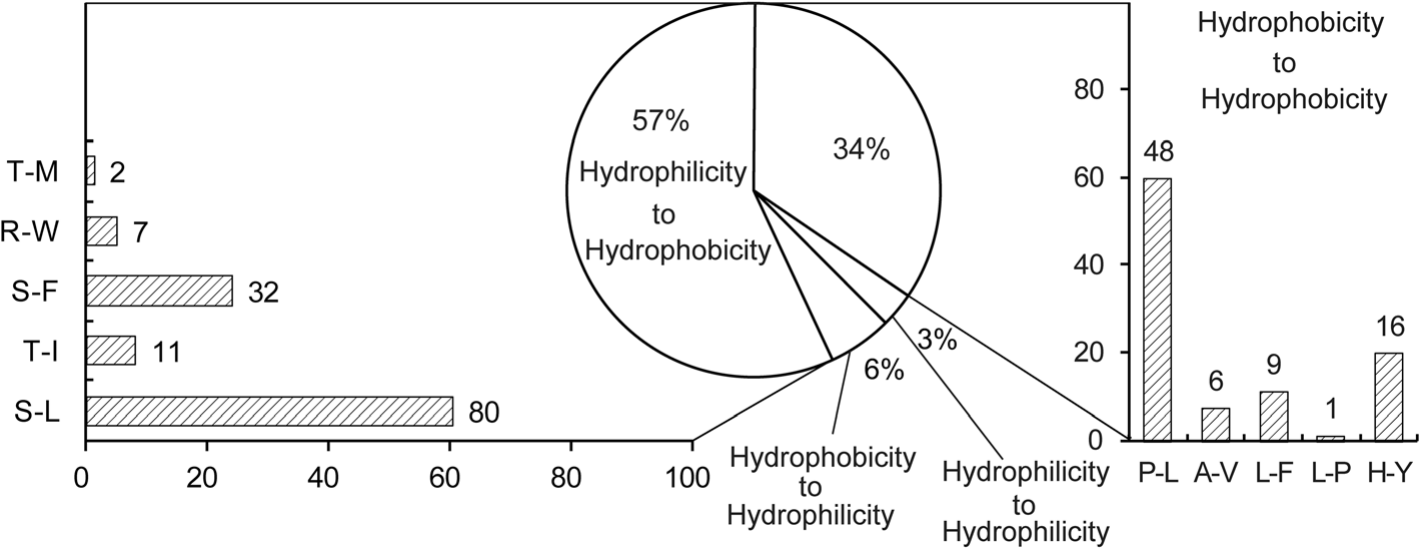
The hydrophilicity or hydrophobicity changes associated with amino acid changes that occurred in non-silenced editing in *Ginkgo biloba* chloroplast transcripts. Hydrophilicity amino acids: T, R and S (Thr, Arg, and Ser, respectively). Hydrophobicity amino acids: A, M, W, I, C, L, V, F, H, P and Y (Ala, Met, Trp, Ile, Cys, Leu, Val, Phe, His, Pro and Tyr, respectively). “-” indicates transformed to

Most RNA editing sites exist in the protein-coding regions and often cause corresponding amino acid alterations. In addition to 16 silent editing sites, there were 239 sites that resulted in corresponding codon changes in *G. biloba*. Among them, 132 editing sites switched amino acids from hydrophilic to hydrophobic, and more than 60.5% of the editing events were serine to leucine, followed by serine to phenylalanine (24.2%) and threonine to isoleucine (8.3%). The amino acids maintained their hydrophobic properties at 80 editing sites, and the highest rate occurred in proline to leucine (60.0%), followed by histidine to tyrosine (20.0%) and leucine to phenylalanine (1.3%). Only 13 and 7 editing sites caused amino acids to change from hydrophobic to hydrophilic and to maintain their hydrophilicity, respectively (Fig. 4).

### RNA editing events in *G. biloba* chloroplast genes may alter protein structures

**Fig. 5:**
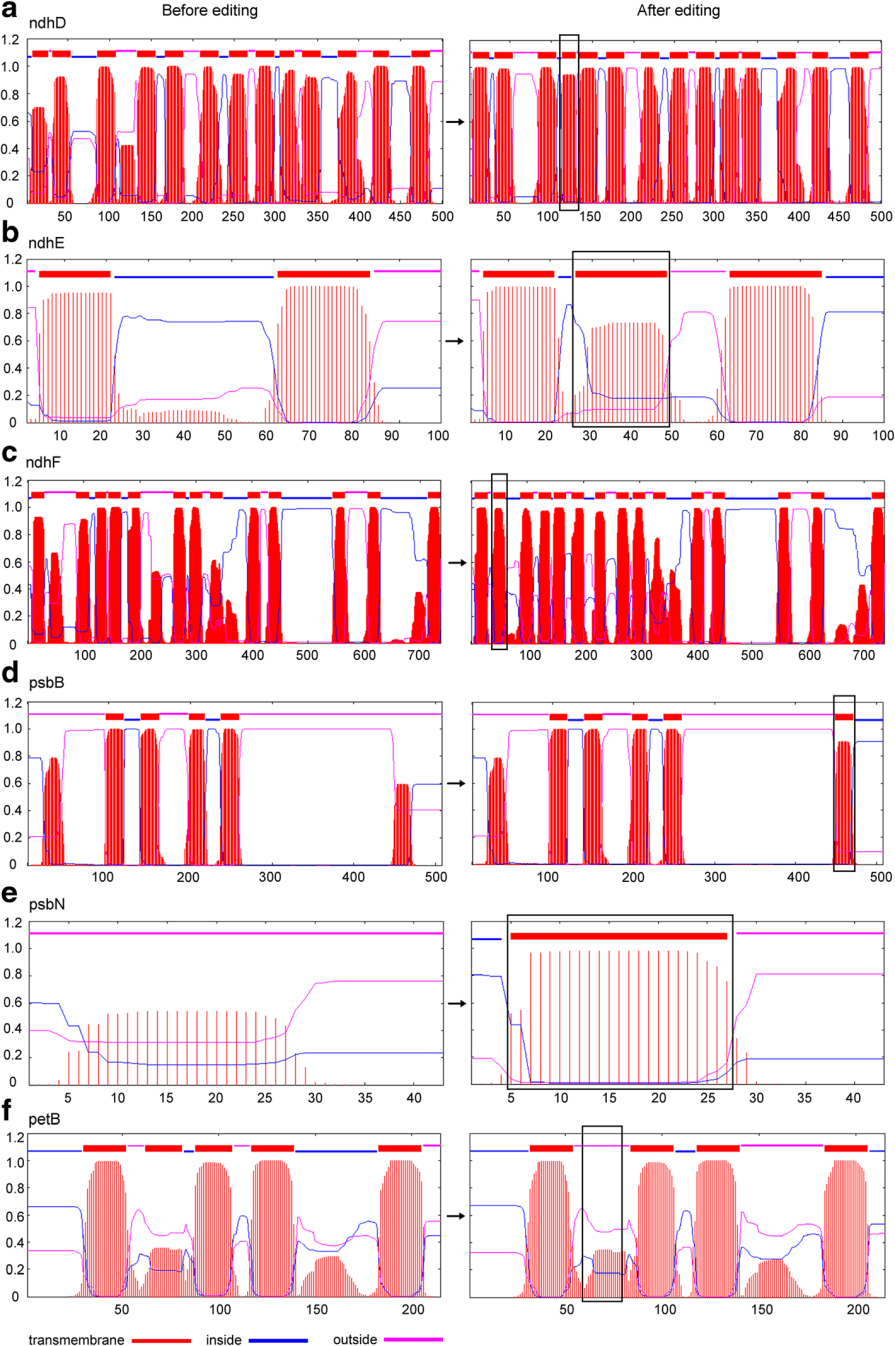
The changes in transmembrane regions after editing. **a** The conversion of S-to-L at ndhD codon position 128 contributes to create a new transmembrane region between codon 113 and 130. **b** The conversion of P-to-L and A-to-V at ndhE codon position 33 and 42, respectively lead to a new transmembrane regions creation between codons 26 and 48. **c** A new transmembrane region at codons 39–61 forms after codon positions 47 (P-to-L), 50 (T-to-I) and 56 (S-to-F) are edited in ndhF. **d** The change of codon position 464 (S-to-F) creats a new transmembrane region between codons 449 and 471 in psbB. **e** An amino acid R-to-C change produces a new transmembrane region between codons 5 and 27 in psbN. **f** The codon position 212 change (P-to-S) results in the disappearance of the transmembrane region that exists in the unedited petB at positions 62–81

In our attempt to understand whether RNA editing affects protein structure, we predicted the secondary structures of 82 proteins before and after editing using bioinformatics software. The results showed that many editing events might change the secondary structures of the corresponding protein. Most editing sites form a new α-helix structure in up- or down-stream regions around the editing codon (Additional file 3: Figure S1). A new cleavage site in the signal peptide within the 18th and 19th codon positions was created in *ndhD*-57 (Additional file 3: Figure S2). Five new transmembrane regions appeared in *ndhD*, *ndhE*, *ndhF, psbB* and *psbN*, respectively, after the corresponding codons were edited (Fig. 5a-e). In addition, a transmembrane region disappeared in *petB* when the amino acid at the 212 codon position changed from proline to serine due to editing (Fig. 5f).

### Comparison of RNA editing sites in different species

A comparison of chloroplast RNA editing events showed that the frequency and type of RNA editing were significantly variable among the major land plant groups, which included 11 angiosperms, 3 gymnosperms, 1 fern, 1 hornwort and 1 moss. C-to-U editing has been widely identified in these land plants, and U-to-C editing has been found only in hornwort and fern. Additionally, *G. biloba* transcripts have the highest C-to-U conversion rate among the three gymnosperms and the number of editing sites is nearly 10 times higher than in other seed plants. During the evolution of plants, the number of editing sites decreased from the highest number, 942, in *A. formosae* to 21–26 in the monocotyledons, *O. sativa*, *S. officinarum*, *T. aestivum* and *Z. mays*. The U-to-C conversions gradually vanished, and the percentage of editing in the second position increased from 58% in *A. formosae,* and 68% in *A. capillus-veneris,* to almost 100% in angiosperms. The silent editing sites decreased. There were 28 and 21 silent editing sites in *A. formosae and A. capillus-veneris*, respectively*.* However, they almost completely disappeared in seed plants (Table 2). The number of start and stop codons created by RNA editing also decreased. Hardly any stop codons were created by RNA editing in angiosperms.

**Table 2:** RNA editing site conditions in higher plant chloroplast genomes

	Moss Hornwort Fern			Gymnosperm			Angiosperm									
	*Pp*	*Af*	*Ac*	*Gb*	*Pt*	*Ct*	*Os*	*Zm*	*Ta*	*Pa*	*Cs*	*At*	*Gh*	*Ab*	*Sl*	*Nt*
Total editing sites	2	942	349	255	26	36	21	26	26	44	32	34	54	35	36	37
C to U	2	509	314	255	26	36	21	26	26	44	32	34	54	35	36	37
U to C	0	433	35	0	0	0	0	0	0	0	0	0	0	0	0	0
1st codon edits	0	165	9	63	0	0	0	0	0	0	0	0	0	0	0	0
2nd codon edits	1	546	237	174	19	30	20	24	24	38	29	29	47	33	33	34
3rd codon edits	0	28	21	14	0	0	0	1	1	0	0	0	1	1	1	1
Silent edits	0	28	21	16	0	0	0	1	0	2	0	0	1	0	0	0
New starts	1	5	21	2	0	1	1	1	1	1	1	1	1	2	2	2
New stops	0	3	3	7	2	1	0	0	0	0	0	0	0	0	0	0
Untranslated region	1	9	ND	ND	ND	ND	1	1	ND	2	0	2	ND	0	0	0

Taxa abbreviations shown above are: *Pp*: *Physcomitrella patens*, *Af*: *Anthoceros formosae*, *Ac*: *Adiantum capillus-veneris*, *Gb*: *Ginkgo biloba*, *Pt*: *Pinus thungergii*, *Ct*: *Cycas taitungensis*, *Os*: *Oryza sativa*, *Zm*: *Zea mays*, *Ta*: *Triticum aestivum, Pa*: *Phalaenopsis aphrodite*, *Cs*: *Cucumis sativus*, *At*: *Arabidopsis thaliana*, *Gh*: *Gossypium hirsutum*, *Ab*: *Atropa belladonna*, *Sl*: *Solanum lycopersicum* and *Nt*: *Nicotiana tabacum*; ND stand for No available data. If there are no special instructions, then these abbreviations apply to Additional file 1: Table S1 and Additional file 2: Table S2

### Evolutionary pattern of RNA editing events in chloroplasts

**Fig. 6:**
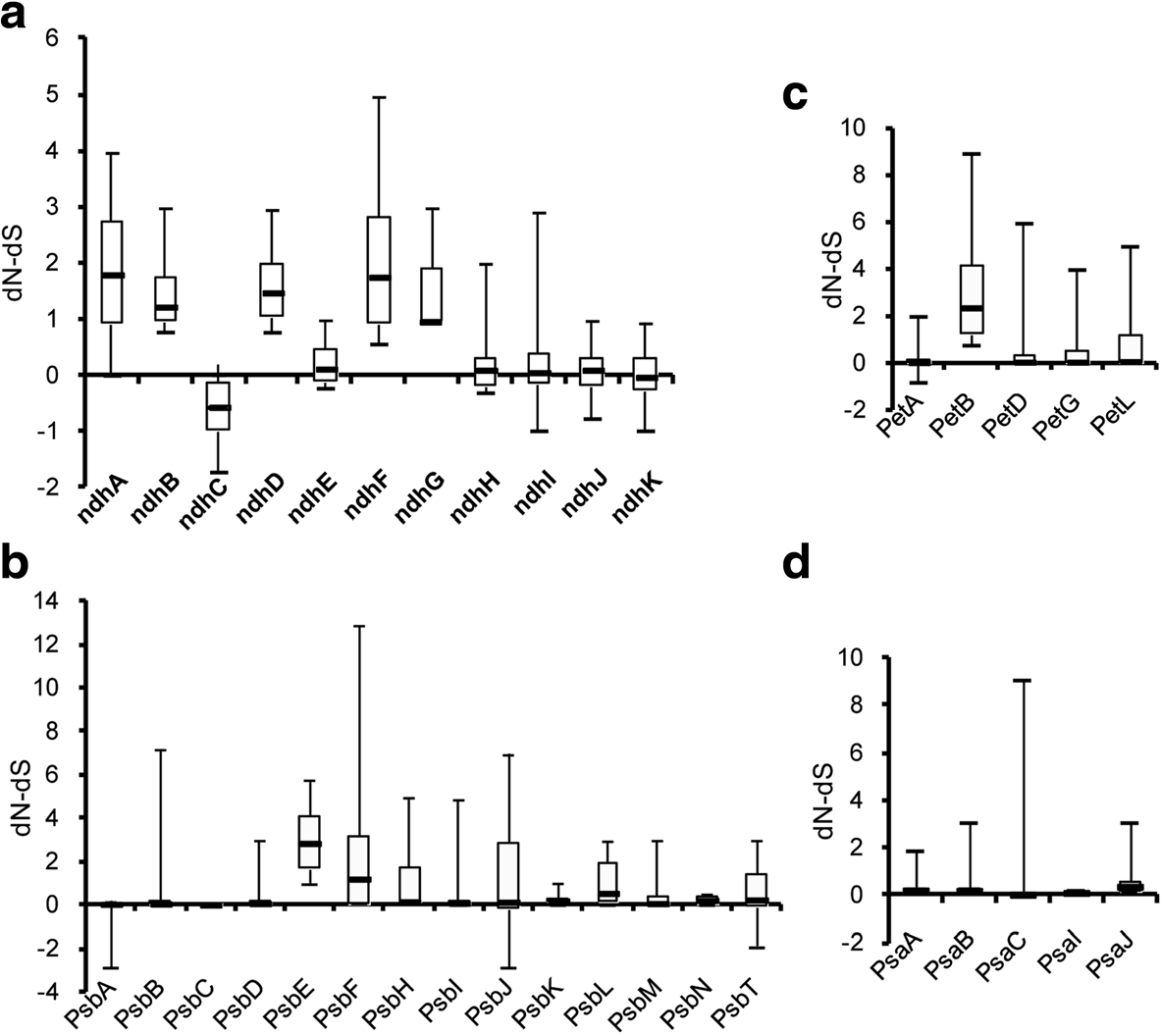
Evolutionary pattern of RNA editing events in four photosynthesis gene families. **a** Evolutionary pattern of RNA editing events in the *ndh* gene family. **b** Evolutionary pattern of RNA editing events in *Pet* gene family. **c** Evolutionary pattern of RNA editing events in *Psb* gene family. **d** Evolutionary pattern of RNA editing events in *Psa* gene family. dN-dS values of DNAs and edited cDNAs with a Z-test for selection were used to analyze the evolution of four photosynthesis-related gene families. Data were obtained from 12 species, *Atropa belladonna*, *Solanum lycopersicum*, *Cucumis sativus*, *Anthoceros formosae*, *Gossypium hirsutum*, *Arabidopsis thaliana*, *Cycas taitungensis*, *Adiantum capillus-veneris*, *Triticum aestivum*, *Nicotiana tabacum*, *Zea mays* and *Ginkgo biloba*

To investigate the evolutionary tendency of RNA editing, the dN-dS values of the RNA editing sites in four photosynthesis-related gene families were calculated using the Z-test of selection in MEGA5.1 Beta software. The dN-dS values of most *ndh* and some *psb* genes were greater than zero (Fig. 6a and b), indicating that these editing sites may have undergone positive selection. The dN-dS values of most of the *psa*, a few *psb* and the *pet* genes studied, except for *petB*, were equal to zero (Fig. 6b, c and d), suggesting that editing sites in these genes may undergo neutral selection. However, we noticed the tendency of the dN-dS values to trend to zero in most *psa*, *psb* and *pet* genes was faster than in the *ndh* gene group (Additional file 3: Figure S3). This occurred because C-to-T point mutations at the genome sites in most of the *psa, psb* and *pet* gene families caused the editing sites to disappear. Moreover, the C-to-U editing at the mRNA level and the reverse mutations at the genome level can both increase codon conservation. For example, *petA*-329, *psaA*-725 and *psbF*-77 were edited in *G. biloba*, but they underwent a reverse mutation to T at the DNA level in *A. thaliana, T. aestivum* and *Z. mays*, causing an increase in the corresponding codon conservation in most of the species (Additional file 3: Figure S4). The results contradicted those of what is commonly referred to as neutral selection, in which mutations are neither beneficial nor detrimental to the ability of an organism to survive and reproduce [36]. In fact, the conservation of amino acids is restored in most of these gene classes due to C-to-T point mutations at the genome level. Thus, C-to-U edits at the mRNA level are unnecessary and even waste energy. As a result, editing sites in these essential genes gradually disappeared during evolution. The evolutionary tendencies of RNA editing in these gene classes acts more like a purifying selection, so, we termed this kind of evolution as ‘similar purifying selection’, in which dN–dS is equal to zero but purifying selection actually occurred to retain codon conservation.

## Discussion

### Abundant RNA editing events are retained in the chloroplast genome of *G. biloba*

**Fig. 7:**
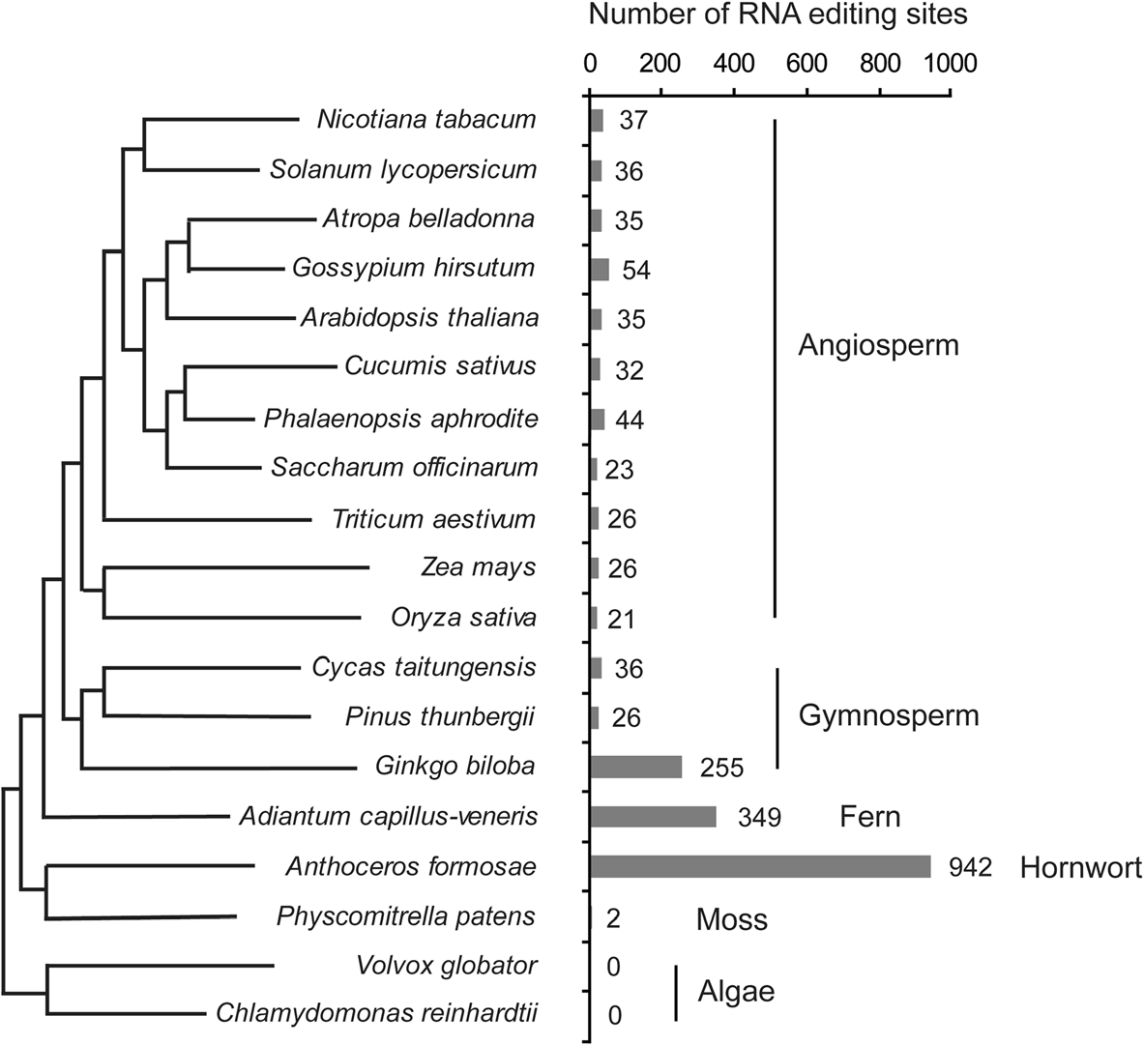
Phylogenetic relationships of 19 species in which RNA editing sites have been reported. This phylogenetic tree was drawn by MEGA5.1 Beta. The number in front of the taxa indicates the number of editing sites in different species (as reported in 2013)

**Fig. 8:**
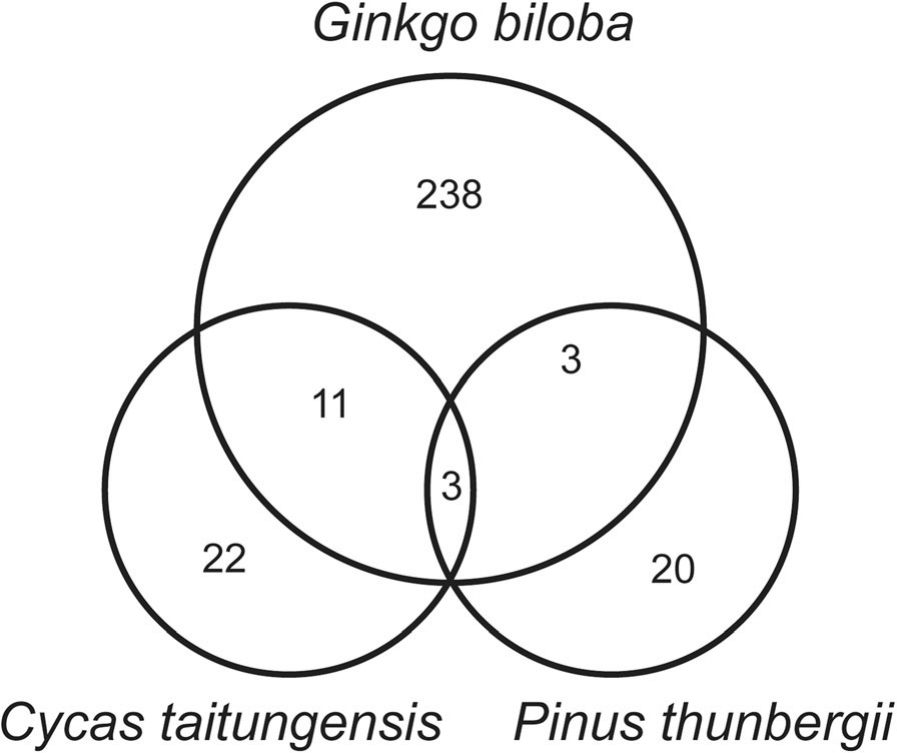
Overview of shared RNA editing site in *Ginkgo biloba*, *Cycas taitungensis and Pinus thunbergii*. The sites present in a given species are enclosed in the respective color-coded circles and the number in the circles indicates the shared or unique editing sites among these species

Except for the marchantiid subclass of liverworts, RNA editing has been observed in the chloroplasts of all of the investigated terrestrial plants. The number of C-to-U RNA editing sites in chloroplasts was variable among plants, ranging from 0 in *Volvox globator* to more than 900 in *A. formosae*. Over 300 chloroplast editing sites were known in early branching land plants, such as *Anthoceros* and *Adiantum*, but fewer sites were edited in angiosperm chloroplast RNAs (Fig. 7). In this paper, we reported that the chloroplast protein-coding transcripts of *G. biloba* contain 255 editing sites, which is by far the highest number of editing sites in a seed plant. A model for the evolution of editing in plant organelles proposed that RNA editing was of monophyletic origin, had a common ancestor with many editing sites during seed plant evolution, and that many of the original editing sites, particularly in seed plants, had been subsequently lost [37]. *G. biloba* is one of the oldest seed plants and appeared in the Early Jurassic period, in which the CO_2_ concentration in the atmosphere may have reached high levels, accelerating climate warming [38]. All of these changes may cause *G. biloba* to acquire many mutations at the DNA level and RNA editing recovered the equivalent genetic information. In addition, comparisons of editing events among three gymnospermaes, *G. biloba*, *Pinus* and *Cycas*, showed that a large number of unique editing sites of *G. biloba* had been lost in *Cycas* and *Pinus* (Fig. 8). *G. biloba* may maintain a more ancestral version of the chloroplast genome than *Cycas* and *Pinus*. Moreover, *G. biloba* shares 11 and 3 editing sites with *Cycas* and *Pinus*, respectively, and three common editing sites, *atpF*-370, *petB*-634 and *psbE*-214, are shared among the three species (Fig. 8). This indicated that the evolutionary conservation of RNA editing is essential for only a few plastid editing sites, which is a common phenomenon among angiosperms and has been verified in many cases [39].

### RNA editing might change the structures and functions of some proteins in *G. biloba*

RNA editing, especially at the second codon position, can alter the encoding amino acid and change the protein primary, secondary or tertiary structures, which might be necessary for the protein function. We analyzed the secondary structures of 82 transcripts in *G. biloba* before and after editing using bioinformatics methods. One editing site changed the signal peptide, eight editing sites could create five new transmembrane regions, and one RNA editing event occurred in *petB*, which caused an existing transmembrane region to disappear. All of the newly created signal peptides and transmembrane regions might play important roles in the localization or formation of the proper spatial structures of the proteins, especially for membrane proteins. Until now, a great deal of experimental evidence supported the view that most of the unedited proteins had lower functional levels than the edited proteins. In peas, unedited acetyl-coA carboxylase carboxyl transferase β is not functional and cannot catalyze the synthesis of fatty acids [40]. In maize chloroplast *rpl2*, the AUG initiation codon generated by a C-U editing of ACG is essential to seed development [41]. In *Arabidopsis*, an editing defect at *atp1*-C1178 has a strong impact on the assembly of the ATP synthase [42]. Of the 255 editing sites in *G. biloba*, two types mainly cause the conversion of amino acids from serine to leucine or phenylalanine and proline to leucine. The former might increase the hydrophobicity of the corresponding peptide and the latter did not change the peptide hydrophobicity, but it could recover the normal curl of the secondary structure or remove misfolding because proline is a helix-breaker.

In addition, the chloroplast genome of *G. biloba* has many partial editing sites. Among the 255 editing sites, 73 partial editing sites were detected. Tseng et al. found that partial editing may regulate plastid gene expression by using a different editing frequency in the non-photosynthetic tissues of *Arabidopsis* [43]. Karcher et al. have found that *ndhB* has different editing profiles in photosynthetic and non-photosynthetic organs in *Z. mays* [44]. Thus, many partial editing sites in *G. biloba* might be associated with different tissues and developmental periods. Further experiments are needed.

### RNA editing may undergo diverse evolutionary patterns in different photosynthetic genes

The RNA editing phenomenon may be a relic of the ancient RNA world that is involved in primordial error correction, such as repairing UV damage or other uncertain factors at the transcriptional level. As a result, RNA editing appears in almost all land plants, except *Marchantia polymorpha* of the marchantiid subclass of liverworts [45]. With evolution, the number of RNA editing sites gradually decreases from the lower to higher plants (Table 2). To understand the evolution of plastid editing sites, we introduced the dN-dS method to predict the evolutionary mode of RNA editing sites. Comparisons of editing sites in the *ndh*, *psa*, *psb* and *pet* genes in 12 plant species revealed that the dN-dS values of *psa*, most of *psb* and the *pet* gene groups were nearly equal to zero (Fig. 6). Additionally, the tendency of the dN-dS values to trend to zero in most of the *psa*, *psb* and *pet* genes was faster than in the *ndh* gene group (Additional file 3: Figure S3). Thus, these genes may undergo similar purifying selection. Most of the genes had an important role in photosynthesis. For instance, the targeted inactivation of *psaI* affects the association of *psaL* with the photosystem I core. Namely, the absence of *psaI* indirectly leads to a defect in photosystem I function [46]. Varotto et al. disrupted the *A. thaliana* photosystem I gene, *psaE*, and observed several defective phenotypes, including a significantly increased light sensitivity and a decreased growth rate of ~50% under normal conditions [47]. Additionally, losing *PsbJ* in tobacco causes the photosynthetic performance to be drastically reduced, as well as an extreme hypersensitivity to light [48]. Salar Torabi et al. also reported mutants in *psbN*-F and *psbN*-R of *N. tabacum* were extremely light sensitive and failed to recover from photo inhibition [49]. Fiebig et al. proposed that essential genes cannot tolerate frequent T to C mutations at the DNA level [50]. For the essential genes, such as *psa*, *psb* and *pet*, most of them have abundant editing sites in ancient species, but many editing sites disappeared during plant evolution due to reverse mutations at the DNA level that restored codons to conserved amino acid residues. Those editing sites were probably essential for the structure and/or function of the encoded protein.

The plastid *ndh* genes encode a thylakoid Ndh complex that purportedly acts as an electron feeding valve to adjust the redox level of the cyclic photosynthetic electron transporters [51]. By far the highest number of plastid editing sites in flowering plants was found in the *ndh* group of genes [52]. In our research, *ndh* genes also possessed the most editing sites and had the highest editing frequency. The *ndh* gene groups might be unessential for plants growing under normal conditions. Burrows et al. hypothesized that the *ndh* complex was dispensable for *N. tabacum* growth under optimal growth conditions [53]. *Ndh* genes are absent in epiphytic plants [54] and are partially lost in *Phalaenopsis, Aphrodite* and *Erodium* [55]. In *P. thunbergii*, most of the *ndh* genes are pseudogenes. Thus, we speculated that the RNA editing sites of the *ndh* genes might be randomly lost and that the loss rate was slow. Therefore, *ndh* genes could keep more editing sites than other gene groups in modern plants. For the *ndh* gene group, we found that RNA editing in *ndhD*, *ndhF* and *ndhG* might create obvious structural changes, which created a new transmembrane region or caused an existing transmembrane region to disappear after editing (Fig. 5). To a certain extent, its occurrence implies that editing in those genes has biological significance. In *Arabidopsis*, the editing deficiency in *ndhF* was associated with a delayed greening phenotype [56]. The decline of the editing efficiency in *ndhB* and *ndhD* affected the flow of cyclic electrons and enhanced disease resistance [57]. Although the products of the majority of *ndh* genes were unnecessary under standard growth conditions, editing was probably most important for the proper function of the NDH protein complex under stress conditions [58, 59]. Due to the RNA editing, *ndh* genes might improve photosynthesis and stress tolerance under harmful conditions, and they may display positive selection during evolution. Some *psb* and *pet* genes, which have a dN–dS bias greater than zero, such as *psbE*, *psbF*, *psbH*, *psbJ*, *psbL*, *psbT*, *petB* and *petL* may have similar evolutionary mechanisms. Thus, RNA editing may be a post-transcriptional regulatory process of ancient genes, as well as part of an evolutionary model with diverse evolutionary directions [60]. We speculated that the editing sites in each gene may undergo diverse evolutionary paths depending on whether the edited codon was important or not for protein executive functions.

## Conclusions

In summary, 255 RNA editing sites were identified in the *G. biloba* chloroplast genome, which is the highest number of RNA editing sites found in a seed plant. Most of the RNA editing sites can restore amino acid conservation, increase hydrophobicity, and even influence protein structures. Using the dN–dS method to predict the evolutionary mode of RNA editing, we found that similar purifying selection constituted the dominant evolutionary force at the RNA editing sites of *psa,* some *psb* and the *pet* groups, and a positive selection occurred in the RNA editing sites of most *ndh*, and a few *psb* and *pet* gene groups.

## Additional files

Additional file 1: Table S1. Primer sequences for detecting RNA editing sites. (DOC 140 kb)
Additional file 2: Table S2. RNA editing sites of *Ginkgo biloba* chloroplast protein-coding genes. (XLSX 26 kb)
Additional file 3: Figure S1. Editing would affect adjacent secondary structures. **Figure S2.** The newly created signal peptide cleavage site. **Figure S3.** Evolutionary tendency of RNA editing sites in 12 species. **Figure S4.** Multiple sequence alignment of *psaA* in 12 species. (DOC 2835 kb)

## Abbreviations

C: Cytosine
CTAB: Cetyltrimethylammonium bromide
gDNA: Genomic DNA
NaAc: Acetic acid, sodium salt
NCBI: National Center for Biotechnology Information
U: Uridine
UV: Ultraviolet

## References

[1] Chateigner-Boutin A-L, Small I (2010). Plant RNA editing. RNA Biol.

[2] Benne R, Van Den Burg J, Brakenhoff JPJ, Sloof P, Van Boom JH, Tromp MC (1986). Major transcript of the frame shifted *coxII* gene from trypanosome mitochondria contains four nucleotides that are not encoded in the DNA. Cell.

[3] Covello PS, Gray MW (1989). RNA editing in plant mitochondria. Nature.

[4] Hoch B, Maier RM, Appel K, Igloi GL, Kossel H (1991). Editing of a chloroplast mRNA by creation of an initiation codon. Nature.

[5] Kugita M, Yamamoto Y, Fujikawa T, Matsumoto T, Yoshinaga K (2003). RNA editing in hornwort chloroplasts makes more than half the genes functional. Nucleic Acids Res.

[6] Wolf PG, Rowe CA, Hasebe M (2004). High levels of RNA editing in a vascular plant chloroplast genome: analysis of transcripts from the fern *Adiantum capillus-veneris*. Gene.

[7] Chen H, Deng L, Jiang Y, Lu P, Yu J (2011). RNA editing sites exist in protein-coding genes in the chloroplast genome of *Cycas taitungensis*. J Integr Plant Biol.

[8] Wakasugi T, Tsudzuki J, Ito S, Nakashima K, Tsudzuki T, Sugiura M (1994). Loss of all *ndh* genes as determined by sequencing the entire chloroplast genome of the black pine *Pinus thunbergii*. Proc Natl Acad Sci U S A.

[9] Guzowska-Nowowiejska M, Fiedorowicz E, Pląder W (2009). Cucumber, melon, pumpkin, and squash: Are rules of editing in flowering plants chloroplast genes so well known indeed?. Gene.

[10] Hirose T, Kusumegi T, Tsudzuki T, Sugiura M (1999). RNA editing sites in tobacco chloroplast transcripts: editing as a possible regulator of chloroplast RNA polymerase activity. Mol Gen Genet.

[11] Jiang Y, Fan SL, Song MZ, Yu JN, Yu SX (2012). Identification of RNA editing sites in cotton (*Gossypium hirsutum*) chloroplasts and editing events that affect secondary and three-dimensional protein structures. Genet Mol Res.

[12] Kahlau S, Aspinall S, Gray JC, Bock R (2006). Sequence of the tomato chloroplast DNA and evolutionary comparison of solanaceous plastid genomes. J Mol Evol.

[13] Schmitz-Linneweber C, Regel R, Du TG, Hupfer H, Herrmann RG, Maier RM (2002). The Plastid chromosome of Atropa belladonna and its comparison with that of *Nicotiana tabacum*: The role of RNA editing in generating divergence in the process of plant speciation. Mol Biol Evol.

[14] Tillich M, Funk HT, Schmitz-Linneweber C, Poltnigg P, Sabater B, Martin M, Maier RM (2005). Editing of plastid RNA in *Arabidopsis thaliana* ecotypes. Plant J.

[15] Zeng W-H, Liao S-C, Chang C-C (2007). Identification of RNA editing sites in chloroplast transcripts of *Phalaenopsis aphrodit*e and comparative analysis with those of other seed plants. Plant Cell Physiol.

[16] Calsa Júnior T, Carraro DM, Benatti MR, Barbosa AC, Kitajima JP, Carrer H (2004). Structural features and transcript-editing analysis of sugarcane (*Saccharum officinarum L*.) chloroplast genome. Curr Genet.

[17] Corneille S, Lutz K, Maliga P (2000). Conservation of RNA editing between rice and maize plastids: are most editing events dispensable?. Mol Gen Genet.

[18] Maier RM, Hoch B, Zeltz P, Kössel H (1992). Internal editing of the maize chloroplast ndhA transcript restores codons for conserved amino acids. Plant Cell.

[19] Binder S, Marchfelder A, Brennicke A (1994). RNA editing of tRNAPhe and tRNACys in mitochondria of *Oenothera berteriana* is initiated in precursor molecules. Mol Gen Genet.

[20] Delannoy E, Le Ret M, Faivre-Nitschke E, Estavillo GM, Bergdoll M, Taylor NL, Pogson BJ, Small I, Imbault P, Gualberto JM (2009). Arabidopsis tRNA adenosine deaminase arginine edits the wobble nucleotide of chloroplast tRNA(Arg)(ACG) and is essential for efficient chloroplast translation. Plant Cell.

[21] Kim B, Kim K, Yang T-J, Kim S (2016). Completion of the mitochondrial genome sequence of onion (*Allium cepa L.*) containing the CMS-S male-sterile cytoplasm and identification of an independent event of the *ccmF*-N gene split. Curr Genet.

[22] Howad W, Kempken F (1997). Cell type-specific loss of *atp6* RNA editing in cytoplasmic male sterile Sorghum bicolor. Proc Natl Acad Sci U S A.

[23] Wei L, Yan Z-X, Ding Y (2008). Mitochondrial RNA editing of F0-ATPase subunit 9 gene (*atp9*) transcripts of Yunnan purple rice cytoplasmic male sterile line and its maintainer line. Act Physiologiae Plant.

[24] Cao Z-L, Yu Q-B, Sun Y, Lu Y, Cui Y-L, Yang Z-N (2011). A point mutation in the pentatricopeptide repeat motif of the AtECB2 protein causes delayed chloroplast development. J Integr Plant Biol.

[25] Deschamps P, Lara E, Marande W, Lopez-Garcia P, Ekelund F, Moreira D (2010). Phylogenomic analysis of kinetoplastids supports that trypanosomatids arose from within bodonids. Mol Biol Evol.

[26] Stoltzfus A (2012). Constructive neutral evolution: exploring evolutionary theory’s curious disconnect. Biol Direct.

[27] Jobson RW, Qiu YL (2008). Did RNA editing in plant organellar genomes originate under natural selection or through genetic drift?. Biol Direct.

[28] Jacobs BP, Browner WS (2000). *Ginkgo biloba*: a living fossil. Am J Med.

[29] Zhou Z, Zheng S (2003). Palaeobiology: The missing link in *Ginkgo* evolution. Nature.

[30] Singh B, Kaur P, Gopichand, Singh RD, Ahuja PS (2008). Biology and chemistry of *Ginkgo biloba*. Fitoterapia.

[31] Lin C-P, Wu C-S, Huang Y-Y, Chaw S-M (2012). The complete chloroplast genome of *Ginkgo biloba* reveals the mechanism of inverted repeat contraction. Genome Biol Evol.

[32] Mower JP, Palmer JD (2006). Patterns of partial RNA editing in mitochondrial genes of *Beta vulgaris*. Mol Genet Genomics.

[33] Tamura K, Peterson D, Peterson N, Stecher G, Nei M, Kumar S (2011). MEGA5: molecular evolutionary genetics analysis using maximum likelihood, evolutionary distance, and maximum parsimony methods. Mol Biol Evol.

[34] Tamura K, Stecher G, Peterson D, Filipski A, Kumar S (2013). MEGA6: molecular evolutionary genetics analysis version 6.0.. Mol Biol Evol.

[35] Goldman N, Yang Z (1994). A codon-based model of nucleotide substitution for protein-coding DNA sequences. Mol Biol Evol.

[36] Nei M, Suzuki Y, Nozawa M (2010). The neutral theory of molecular evolution in the genomic Era. Annu Rev Genomics Hum Genet.

[37] Tillich M, Lehwark P, Morton BR, Maier UG (2006). The evolution of chloroplast RNA editing. Mol Biol Evol.

[38] Delannoy E, Fujii S, Colas C, Brundrett M, Small I (2011). Rampant gene loss in the underground orchid Rhizanthella gardneri highlights evolutionary constraints on plastid genomes. Mol Biol Evol.

[39] Pieńkowski G, Hodbod M, Ullmann C (2016). Fungal decomposition of terrestrial organic matter accelerated Early Jurassic climate warming. Sci Rep.

[40] Sasaki Y, Kozaki A, Ohmori A, Iguchi H, Nagano Y (2001). Chloroplast RNA rditing required for functional acetyl-CoA carboxylase in plants. J Biol Chem.

[41] Bock R (2000). Sense from nonsense: How the genetic information of chloroplastsis altered by RNA editing. Biochimie.

[42] Hammani K, Colas des Francs-Small C, Takenaka M, Tanz SK, Okuda K, Shikanai T, Brennicke A, Small I (2011). The pentatricopeptide repeat protein OTP87 is essential for RNA editing of nad7 and atp1 transcripts in Arabidopsis mitochondria. J Biol Chem.

[43] Tseng CC, Lee CJ, Chung YT, Sung TY, Hsieh MH (2013). Differential regulation of *Arabidopsis* plastid gene expression and RNA editing in non-photosynthetic tissues. Plant Mol Biol.

[44] Karcher D, Bock R (2002). The amino acid sequence of a plastid protein is developmentally regulated by RNA editing. J Biol Chem.

[45] Steinhauser S, Beckert S, Capesius I, Malek O, Knoop V (1999). Plant mitochondrial RNA editing. J Mol Evol.

[46] Horváth EM, Peter SO, Joët T, Rumeau D, Cournac L, Horváth GV, Kavanagh TA, Schäfer C, Peltier G, Medgyesy P (2000). Targeted inactivation of the plastid *ndhB* gene in tobacco results in an enhanced sensitivity of photosynthesis to moderate stomatal closure. Plant Physiol.

[47] Varotto C, Pesaresi P, Meurer J, Oelmuller R, Steiner-Lange S, Salamini F, Leister D (2000). Disruption of the *Arabidopsis* photosystem I gene *psaE1* affects photosynthesis and impairs growth. Plant J.

[48] Hager M, Hermann M, Biehler K, Krieger-Liszkay A, Bock R (2002). Lack of the small plastid-encoded *PsbJ* polypeptide results in a defective water-splitting apparatus of photosystem II, reduced photosystem I levels, and hypersensitivity to light. J Biol Chem.

[49] Torabi S, Umate P, Manavski N, Plöchinger M, Kleinknecht L, Bogireddi H, Herrmann RG, Wanner G, Schröder WP, Meurer J (2014). *PsbN* is required for assembly of the photosystem II reaction center in *Nicotiana tabacum*. Plant Cell.

[50] Fiebig A, Stegemann S, Bock R (2004). Rapid evolution of RNA editing sites in a small non-essential plastid gene. Nucleic Acids Res.

[51] Sazanov LA, Burrows PA, Nixon PJ (1998). The plastid ndh genes code for an NADH-specific dehydrogenase: Isolation of a complex I analogue from pea thylakoid membranes. Proc Natl Acad Sci U S A.

[52] Maier RM, Neckermann K, Igloi GL, Kössel H (1995). Complete sequence of the maize chloroplast genome: gene content, hotspots of divergence and fine tuning of genetic information by transcript editing. J Mol Biol.

[53] Burrows PA, Sazanov LA, Svab Z, Maliga P, Nixon PJ (1998). Identification of a functional respiratory complex in chloroplasts through analysis of tobacco mutants containing disrupted plastid *ndh* genes. EMBO J.

[54] Funk HT, Berg S, Krupinska K, Maier UG, Krause K (2007). Complete DNA sequences of the plastid genomes of two parasitic flowering plant species, *Cuscuta reflexa* and *Cuscuta gronovii*. BMC Plant Biol.

[55] Chris Blazier J, Guisinger MM, Jansen RK (2011). Recent loss of plastid-encoded *ndh* genes within Erodium (Geraniaceae). Plant Mol Biol.

[56] Bentolila S, Heller WP, Sun T, Babina AM, Friso G, van Wijk KJ, Hanson MR (2012). RIP1, a member of an *Arabidopsis* protein family, interacts with the protein RARE1 and broadly affects RNA editing. Proc Natl Acad Sci U S A.

[57] García-Andrade J, Ramírez V, López A, Vera P (2013). Mediated plastid RNA editing in plant immunity. PLoS Pathog.

[58] Pineau B, Bourge M, Marion J, Mauve C, Gilard F, Maneta-Peyret L, Moreau P, Satiat-Jeunemaître B, Brown SC, De Paepe R, Danon A (2013). The importance of cardiolipin synthase for mitochondrial ultrastructure, respiratory function, plant development, and stress responses in *Arabidopsis*. Plant Cell.

[59] Rumeau D, Peltier G, Cournac L (2007). Chlororespiration and cyclic electron flow around PSI during photosynthesis and plant stress response. Plant Cell Environ.

[60] Hirose T, Wakasugi T, Sugiura M, Kössel H (1994). RNA editing of tobacco *petB* mRNAs occurs both in chloroplasts and non-photosynthetic proplastids. Plant Mol Biol.

