# Supplementary material for: Abundant RNA editing sites of chloroplast protein-coding genes in Ginkgo biloba and an evolutionary pattern analysis
Source: BMC Plant Biol. 2016 Dec 1;16:257. doi: 10.1186/s12870-016-0944-8 (PMC5131507; doi:10.1186/s12870-016-0944-8)
Supplement: Additional file 1: Table S1. — Primer sequences for detecting RNA editing sites. (DOC 140 kb) [file 12870_2016_944_MOESM1_ESM.doc]

**Additional file 1: Table S1 Primer sequence for detecting RNA editing sites**

| Gene | Forward primer sequence （5’→3’） | Reverse primer sequence （5’→3’） |
| --- | --- | --- |
| *ycf1* | CGGTTGAATTTACATCTGCTC | AGTCTATTTACGGGGTGCAG |
|  | CTACTCACCTATCCATGCAG | GAAAAATCCCCATCTTCTCC |
|  | TCCTGTAAAGACCGAAGTGT | AATCTTTCAGCGGATCTTTC |
|  | TTGTGAGATCGATATGAAGG | GATCTATCCCCGAAAGAAGC |
|  | TGTACATTCCCCTTTCTTTC | TGTCCAATCATCTAGATCTTTC |
|  | GAATGAATGATCAGATCCCC | CTTGCGGTTCTTTAGAATCC |
|  | CGAACATCGTATCCTTTTATC | CTATCTTCCTGATCCCGAAC |
|  | GATGATCATACCGAGAAAATC | TCATTCTCCAGATTGAAACG |
| *ycf2* | GAAGTCCTTGAATCGATTTAAG | CACAAAAAGCAGACATGAGG |
|  | GATCAACTTGAACTCGAAGC | GAGAAGAAAGATCCCAAAGA |
|  | GCGAATGGATTTTGACAAAC | TTTTCAAAATGCCCATCAAT |
|  | GAAAACCCTCTTAACAATTACAG | TTGACCGGTGCTGATGTGAA |
|  | AGAACACTGATTTTTCGACG | TTCTTAACACGTTCATTGAAAC |
|  | GAAAATGCTTCATCGAATTC | TCCATGCTGAAGCACGATCT |
|  | ACATATCCATCTATTTTCTGAAC | CTGCCGTTGTGAGATAGGAA |
|  | AGTGGATCCAGCTTCAATAG | AATAAAACCCCTTTCGCATC |
|  | GGAATATGTCGAGGTAAGTCTG | TGAATTGGTCATGAATTTTG |
|  | GCTTTTTCTAGGAAGACGATTT | AATTCCATTGCTCTTTCATC |
| *ycf3* | TTYGTTCRTGGGATCMTTTCG | GAATTCCGTTTCTTTCTCCC |
| *ycf4* | CCGAAGAAACAAATTTTTGG | AGATCTATCTATTCGGATGTCG |
| *ycf12* | GTGGAAAACGATTGATCTTG | CCCGAACACTCAATACTTG |
| *psaB-*  *psaA* | GTTGGCGGGTTATTCTTATG | TTCATGAGGAAGTGGTATCTC |
|  | CTCTATCTTGGGCAGGACAC | CTGAAAACATATCTTGAGGACG |
|  | GTATCACACCTCAACTGGGC | GGCTGGATARCAGGAGCRAC |
|  | TTAGGTGCTCATTTTGTTTG | CAAGCCAAAGAACTKACTCC |
|  | GGAARCCRAGTGTTTCATGG | ATAAACTAGCCCAACTCAGATG |
|  | CATTATGACAGGGGCMTTTG | AGTTCYTGCCAATATCCACG |
|  | CAATCCTTTYGGTATGAACAG | TATATAGGGATAGGGGGACTT |
| *psaC* | TTGACATCCTAACTAATGGG | ATCTTGGGTTGAGTATGGG |
| *psaJ-*  *rpl33-*  *rps18* | TCATACACGTACCCTAATCG | GATCGAATAGGTGTATTGCG |
|  | CAAGGGTGGAGATGTAAGAG | TCTAATTGAGATCTTCATTTTG |
| *psaI* | TCATTCCTTGTAGTAGAGATC | CCATTTATGAGACCTGATCC |
| *psbA -psbT* | GCGAGAAAGTTACATAGTGTC | ACATGGTTCCAGCGACAAT |
|  | CCATCTTAGTGTTCGTCCTC | GYCCAAAAGTAAACCAACCC |
|  | TGCTAGACGYGCTCAATTAG | TTCGATGAAAGAAGTGGGAG |
| *psbB* | GCGAGAAAGTTACATAGTGTC | ACATGGTTCCAGCGACAAT |
|  | CCATCTTAGTGTTCGTCCTC | GYCCAAAAGTAAACCAACCC |
|  | TGCTAGACGYGCTCAATTAG | TTCGATGAAAGAAGTGGGAG |
| *psbC-*  *psbD* | GTCCGTAGATGATCAAACCG | CGTTTATTGGAAAAAGCAACCCC |
|  | GGGAGTTGCCGGAGTATTG | CCCACCAGGACCTACTCCC |
|  | CCGGATTAATTGTATTCTGGG | AAYGCTTGAGCTTGAGAGGC |
|  | GCTGTGTTTGGTTTCATTGC | GAGGGGGAGTCTTTAAGGATAC |
| *psbE-*  *psbF-*  *psbL* | TCCTRATCTCTATCTTTYATGGG | GRATCCTTCCAGTGGTATCG |
| *psbH* | CAAAGTTARCAGATCTCAACC | CACGATCRAACTACCGGAAC |
| *psbI* | TACACTTCCAATACTGATCCC | TCTCTTTCTGATTTTTTGTTTG |
| *psbJ* | CAAAATGTTGAATTGAATCG | TCGCATGTGTAGTATTCCTC |
| *psbK* | GCTTTATCTCCATAATGTTGATTC | AATTTGTCATTTGTCTCCCC |
| *psbM* | GATCAAATCTCGAGTTATTGTAG | TATTCCAGACTTATGATTCGG |
| *psbN* | GGTTGAGATCTGYTAACTTTG | TCATCGAAAGAATGACCTTC |
| *psbZ* | ATGGGGATCGGATGGTATAG | CCCKTCVGAACTAKAATGCC |
| *clpP* | CATGAACATGTCCTAGAAGTC | ATATCTTCTCTTTCCCCTGG |
| *ndhA* | AGGCTTATTACGCACAATCC | CCTATTGCAACTCCCAATAG |
|  | ATTATGGGAGAGGTTGATCG | ACCATATCTRGACTGTGCYTC |
|  | GCTGCTCAATCTATTAGTTACG | GAGAACATGTGRTAAATATCCC |
| *ndhB* | AGATTGVWTGAAGTTACTAATTC | AAGCTGAAGCAGCTACTTTC |
|  | CATCAATGGACYCCYGAC | TCCAGTTAGTAAGAAGAATCC |
| *ndhD* | GCAGYATGGGTCTAGCTTACT | CATACATGTACTATAATGCGCT |
|  | ACCTGGYTACCRGATACTCATG | AATTCGATCATACTCCGCAG |
| *ndhE-*  *ndhG-*  *ndhI* | CAAAAATGATCTATTCAGGG | GGTATGGTCCCTATTAATCG |
|  | CCRATATCGGTGATTGRAGA | GATAAATGARCCCCAATACG |
|  | CCTGAACAYATCATGGTYCG | GTATTTACAATCGATCCGGG |
| *ndhF* | GAATAATGATTCGATCTTCCC | TTCCATAGCATCAGGTAACC |
|  | CCTGAACAYATCATGGTYCG | GTATTTACAATCGATCCGGG |
|  | TAAYCGTGYAGGGGATTTYG | AAGTTRTTCCTGTAATTGGC |
|  | GATCAGTGATYCATTCYATGG | TYGMTGGAGTYARCCATTGAG |
|  | AAGAATCGGRCAATMYAATGC | TACAGGGGATAATGGATCAG |
| *ndhH* | GTCAATTARGTATTCGTGGRC | ATCCCATTGTATTCCAGAAG |
| *ndhJ-*  *ndhK-*  *ndhC* | TTTATCYATTTCACAGGAGTC | CTGTAATAGTACATGCTCCC |
|  | GATCTAGTCCTAGACAAGCC | GCTATAGAGGCCCAATCYTC |
|  | CTAGCCAAGCATAAGTTAGC | ATTCCTCAAATTGAAGATCTC |
| *petA* | GTAACTRTGATAGYTACCTACCYC | GTATTGTTGCTCTTGCTYCC |
|  | TGTAATAGGTCCTGTTCCYRGT | TTGCATCAGTCATCAATGAG |
| *petB* | GTTCCGGTAGTTYGATCGTG | ACTCCCATAATCCATHTTCTCC |
| *petD* | GGTATTTCAGGYCCTTTATAGAG | TGAARCAAYKACTCCCTAGATAT |
| *petL-*  *petG* | CTTAGGTAATTGCCTCATAAAG | CCCAGRATAAGTAGGAGGYCGGAGGYC |
| *petN* | GTKATTAGTTCCCTTATCATCRG | TCATTMCKGAATGATTTRGAAC |
| *atpB-*  *atpE* | CATTTTTGGTAGGATTAGCG | CTGACCATAGACCAGAGCTAC |
|  | AACGTACTCGTGAAGGAAATGATCT | CCATCTAATTCTCCRGAAAGG |
|  | ACAACCCTTTTTYGTAGCAG | TGAAATATAGAATCGATTTCCG |
| *atpF-*  *atpA* | GGAACTGGAACAAGAGATAGAG | TCRTCGGGTCGAATRGTTAC |
|  | CTYAGAGCCATGAAAAACAYAAC | CTACCTGAGCYACRGAAGARGC |
|  | TTCTATGATCCCTATAGGRCG | CGGATCCTACTCTRGATACRG |
|  | TGARACTCAAGCTGGRGATG | GATAGACTCTTCAGCTCTTCC |
| *atpH* | CGCATTATCAACCATTTCTC | AYGCTCTCCCCTYATARATAAG |
| *atpI* | GAGGAATCAGTCATTCAACC | TTTCGCTCCTACCATACATC |
| *rpl14* | CAAGAAGAAGATTCCTAGTTCG | TTTTTTACYATGTCAGCGTTTC |
| *rpl16* | ACYATYTATGGAGTATTGGGG | TTGTCCGCYACATTYAAATAAG |
| *rpl20* | CTCAATGAACCAAATGAAGG | TCAATGTCTCTACCTTCCC |
| *rpl22* | GTCGTTAATTAGGAGGTGC | CTGTCCCATACATATCTTCC |
| *rpl32* | GTAAACATATGTCTTTCACATCG | TTTTTCTCATTGCTGCTCTC |
| *rpl36-infA* | AGGGAAAAGTCAGTTGGATG | TTTTTCATTTTGACATTCGG |
| *rpoA* | CGTGAYGTAACYCCTATGCC | GGTTTACAMCGATAACTTGG |
|  | AGATGCTGTMTYTATGCCTG | GAAAAAATTCGAGGAAGGGG |
| *rpoB* | AGACTCTTATTATGGACGGG | CTCRTTCTCGGGTATATCAAG |
|  | GGCGATTTGGTATTTTCCGAG | GTATTCATCTTCTCCCGGTG |
|  | CTATTCATGCAAAGATTGGTC | CATCACCYGKTTSTAYCCAAG |
|  | GCCCTGAGAGAATCACTAAAG | TTCCTRTTGTAACRGGTTGTTC |
|  | CAGCYAATCCATGGGTATTTG | ACCAAGCRCAAATYTKCTCRG |
| *rpoC1* | CAGATAGATARGAAGGAAGTTTG | CACTTAATTGAACGATTGGTC |
|  | CAVAATGGATGGTYYTATGC | AAACATRAGTAAACGVGCTTC |
|  | GTGSRGGATTTAATGCGGAC | ATTCAGCAATGAGTTCAAGC |
| *rpoC2* | ARATMGAGATYGAGGAAGCC | TTCAACAAGTCTRCGCGTG |
|  | CGCGAAGGACTYTCTTTRAC | ATAACTTGTTCCGATTCCAC |
|  | ACACGTCAYGGRCATCCTG | ATGAAAAGRAAGCGACCTCC |
|  | AGTTCAGGAATTMTMAAATATGG | CTTACTAAAGAATTGACTACCCC |
|  | ATTAGYTTGRTGAAATACCCC | GAACGRGCTTCTGAYAATTG |
|  | CTGTTCATRGTCATTATGGRG | AGAGATCGCCTGTTCATATC |
| *rbcL* | GCAGACCTCGTCCTTGYAAG | TCRCATGTACCYGCAGTAGC |
|  | TRAATTCCCAACCATTYATGC | TAGTAAAAGATTGGGCCGAG |
| *rps2* | AGATGTAAGAAATTCCGCC | ACGAGATCTAGGAGTAGTAGCC |
| *rps3* | AAAGATCCAGTGGAAGGAAG | GACAGATCATGGAATCATTTG |
| *rps4* | CTATTCCCCTTTCCAGTACC | GTCCAGATGTCCAGTAACCC |
| *rps7* | CAATATGGAGTCAAAAAGCC | TCTCTCCCTAATCGATCAAG |
| *rps8* | AAATAGTYYCATTGGCTCCC | TTTCCCTTTCATAGAGATATCC |
| *rps11* | TGAAAAATGTCTTTTGAGAG | TGATATTTCATCCCGAATC |
| *rps12* | CGATATGGTTAGGATTAATCTG | AATCTCTCAGTCTTTTGAAGC |
|  | GTTTGGAGGGAGATCTTTC | CTAGCAATTAACATGGCATC |
| *rps14* | CAATCCTTTYGGTATGAACAG | TATATAGGGATAGGGGGACTT |
| *rps15* | TTCTTCCGACCATGAATC | GCTCATTAAGATCCGGAAT |
| *rps16* | CAAATTAGATTCTCGTTCCTATC | GGATGGTGAAAGAACAATTG |
| *rpl2-rps19* | ACTATAATATCGATCAATATGGG | CCCCTTCCAAGTGTTATTTC |
|  | CCCGAGAGCTCCTATATCAG | TCTTCATCGACTTTTACCCC |
| *chlB* | ATATCTCTGTGATGTGGGG | CCAATTAGATTTGCAGAAGG |
|  | CGTAATTTTTGCAGATGTGG | ACACGAATTCCCATCTCTC |
|  | CTACATCGATGGACAAACTC | TGGAATCTATTTTCGGAATG |
| *chlL_chlN* | AATAACGGGAGATTGGAAG | TACCCTTGACTCTAGATACTCG |
|  | TTAGTCGGAAATCGTACATC | ATATAATTACGCTGGGATCTC |
|  | GGCAGAATTAGAAGAAGGAG | AAATCCGTTCTTCCCTTTC |
|  | TGGTAAGACGTGATAAATGTG | TTCGATCTGAAAGTATTGGG |
| *accD* | ATTGCAAAGTAATGAGAGATCTC | AACTGAAACTTCCYTCTTGC |
|  | GTATGGGCTCSGTAGTAGGTG | AATTATTCCGCCTTTCGCTG |
| *matK* | GTCCYRACYAGATCGCACC | GTACCCTTCAGAGCYATAAKRG |
|  | TATCACGATCTGTTCTCCAC | CGAATCGATCAAGAATATCATC |
|  | CAATAGCTCCGATTATACCTC | CTGGCAATTGCTCAATAAGC |
| *cemA* | CGAAGGATATTCTTTCTCAG | ATATTCCCACTCAATCGTTG |
| *ccsA* | GTAGATTTATCCTATCGATCC | GAAAAAGTGTCTTGCAAATC |
|  | TATGATATTGCTTGGCTATG | AATTCCATATAATGACTATGTAGT |
