# Supplementary material for: Abundant RNA editing sites of chloroplast protein-coding genes in Ginkgo biloba and an evolutionary pattern analysis
Source: BMC Plant Biol. 2016 Dec 1;16:257. doi: 10.1186/s12870-016-0944-8 (PMC5131507; doi:10.1186/s12870-016-0944-8)
Supplement: Additional file 3: Figure S1. — Editing would affect adjacent secondary structures. Figure S2. The newly created signal peptide cleavage site. Figure S3. Evolutionary tendency of RNA editing sites in 12 species. Figure S4. Multiple sequence alignment of psaA in 12 species. (DOC 2835 kb) [file 12870_2016_944_MOESM3_ESM.doc]

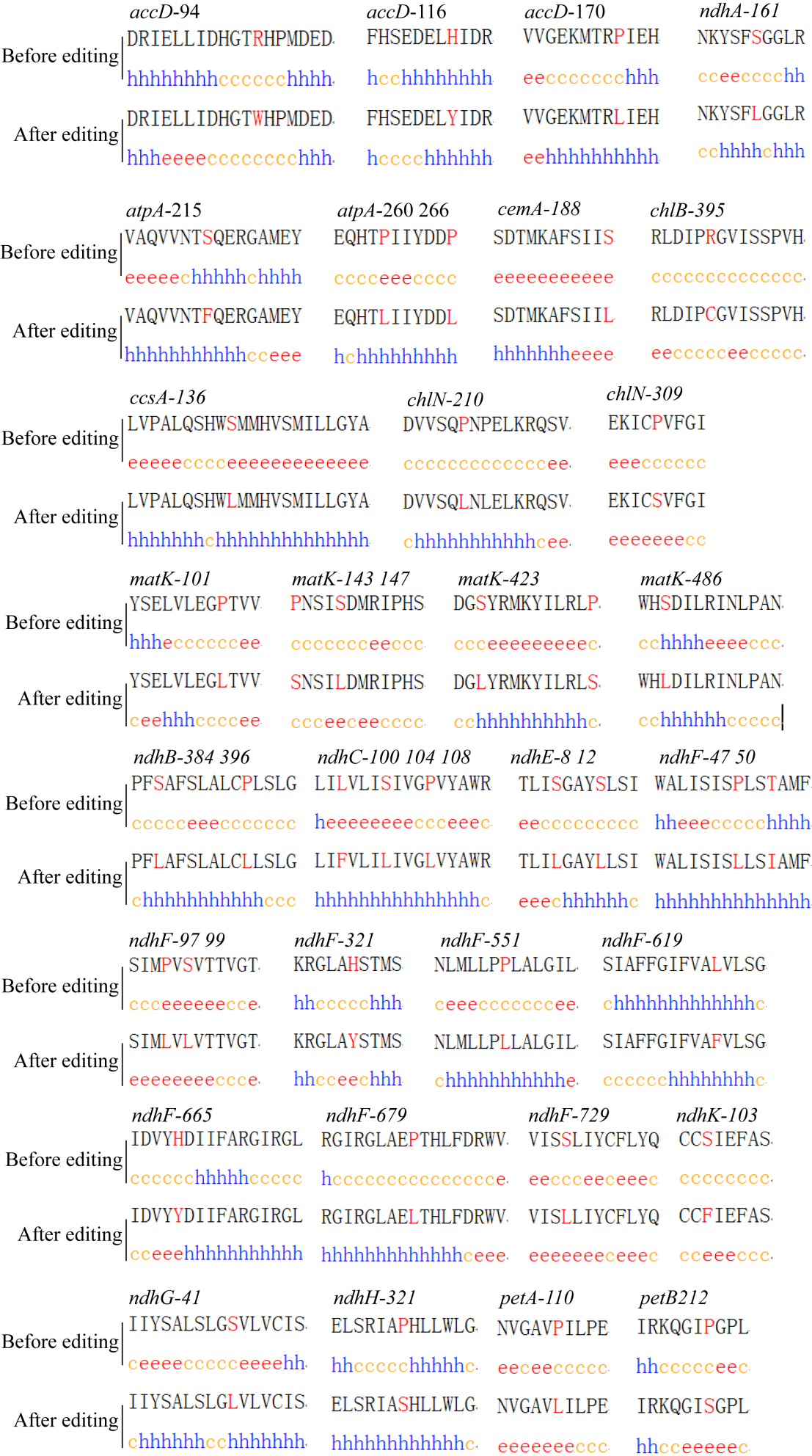


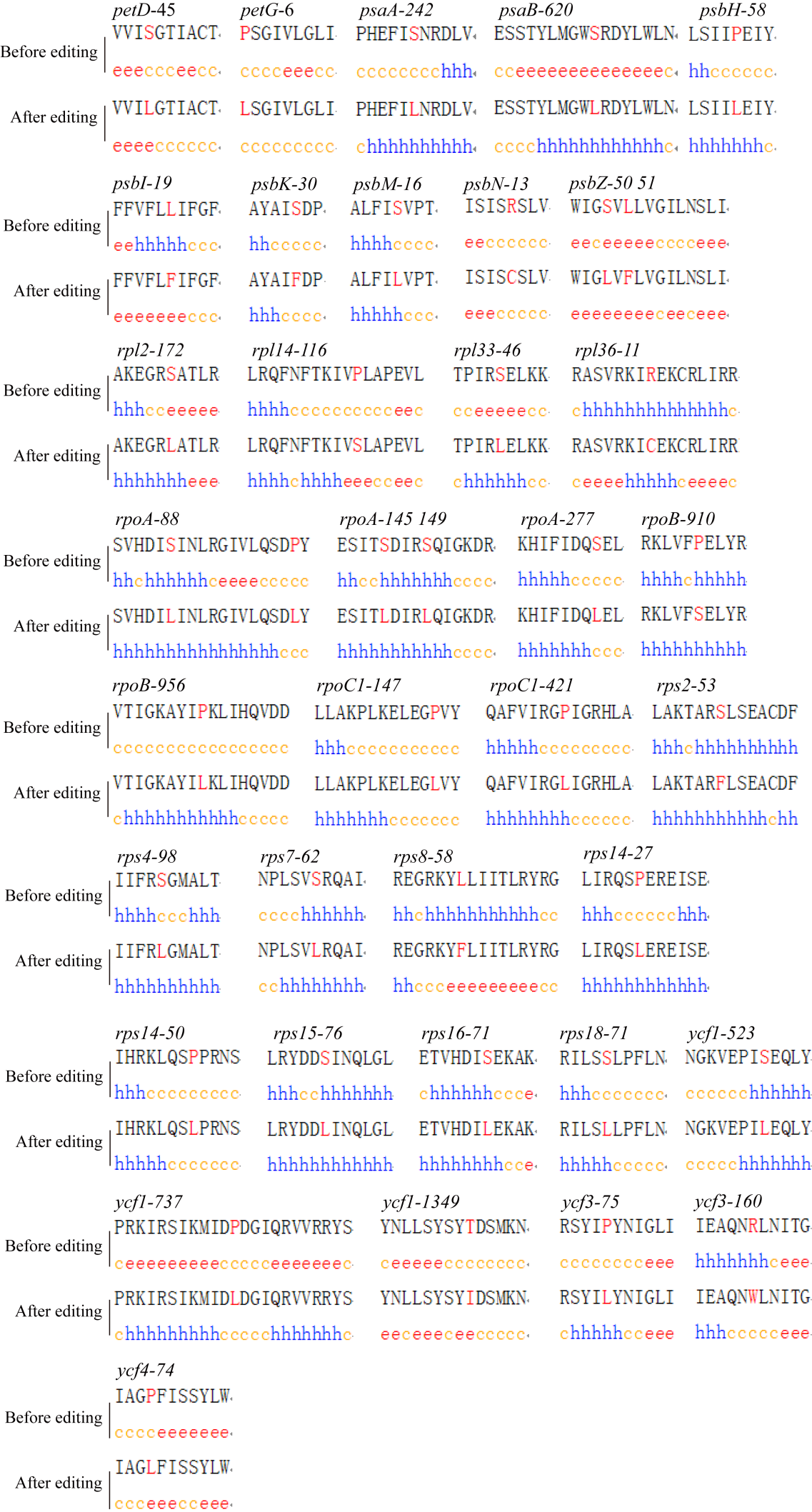


**Additional Figure 1. Editing would affect adjacent secondary structures**

The red capital letters indicate the edited amino acids; red lowercase letters indicate the changed secondary structures.


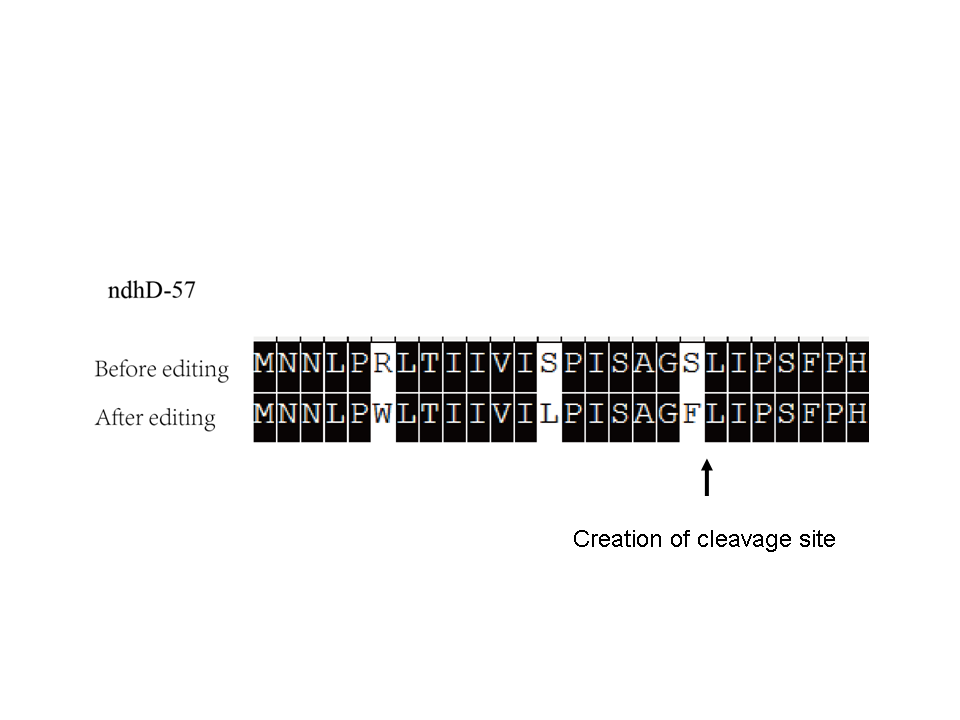


**a**


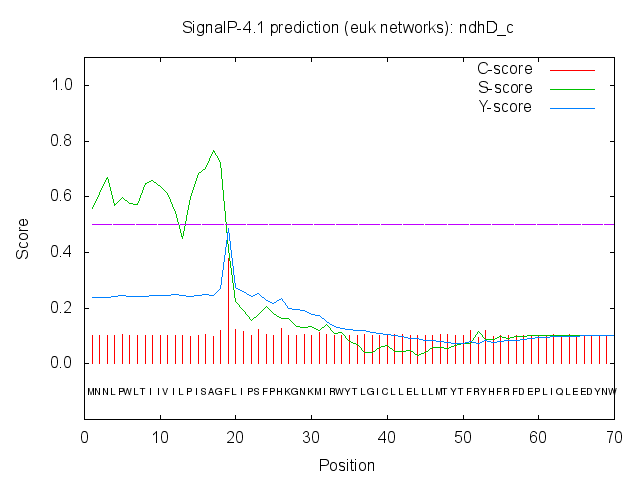

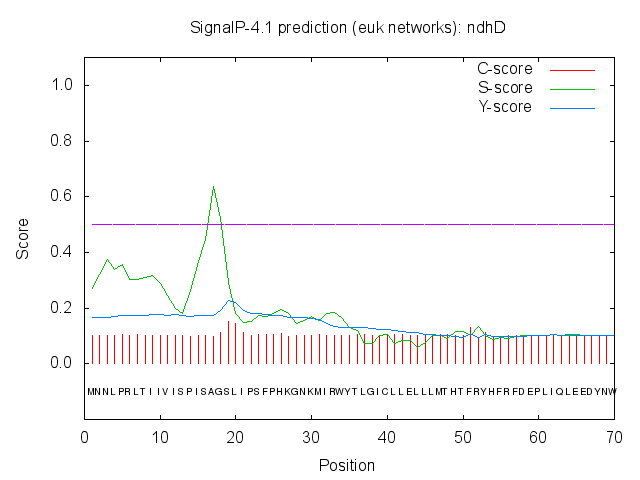


**b**

**c**

**Additional Figure 2. The creation of a new signal peptide cleavage site**

(a) Alignment of the amino acid sequence before and after editing.

(b) Prediction of ndhD signal peptide before editing.

(c) Prediction of ndhD signal peptide after editing.


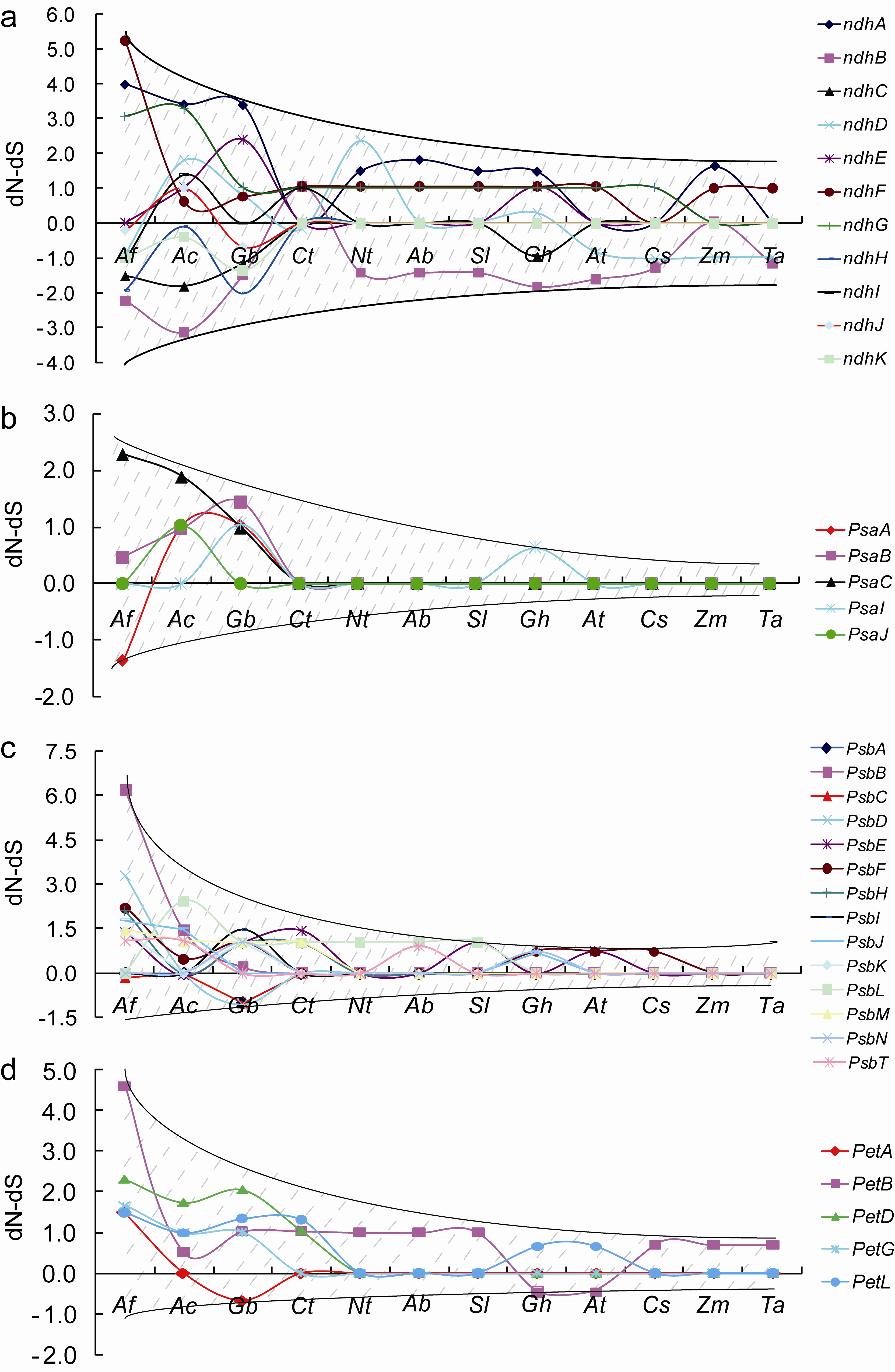


**Additional Figure 3. Evolutionary pattern of RNA editing events in 12 species**

(a) The selection mode of *ndh* genes

(b) The selection mode of *psa* genes

(c) The selection mode of *psb* genes

(d) The selection mode of *pet* genes

The species on X axis is arranged as Kronaqwest classification system from lower plant to relative higher plant. To see the visual evolutionary trends of editing sites, data point line was used to show the overall trend of selection pressure on ndh grourp gene. dN-dS value is computed between DNA and cDNA.


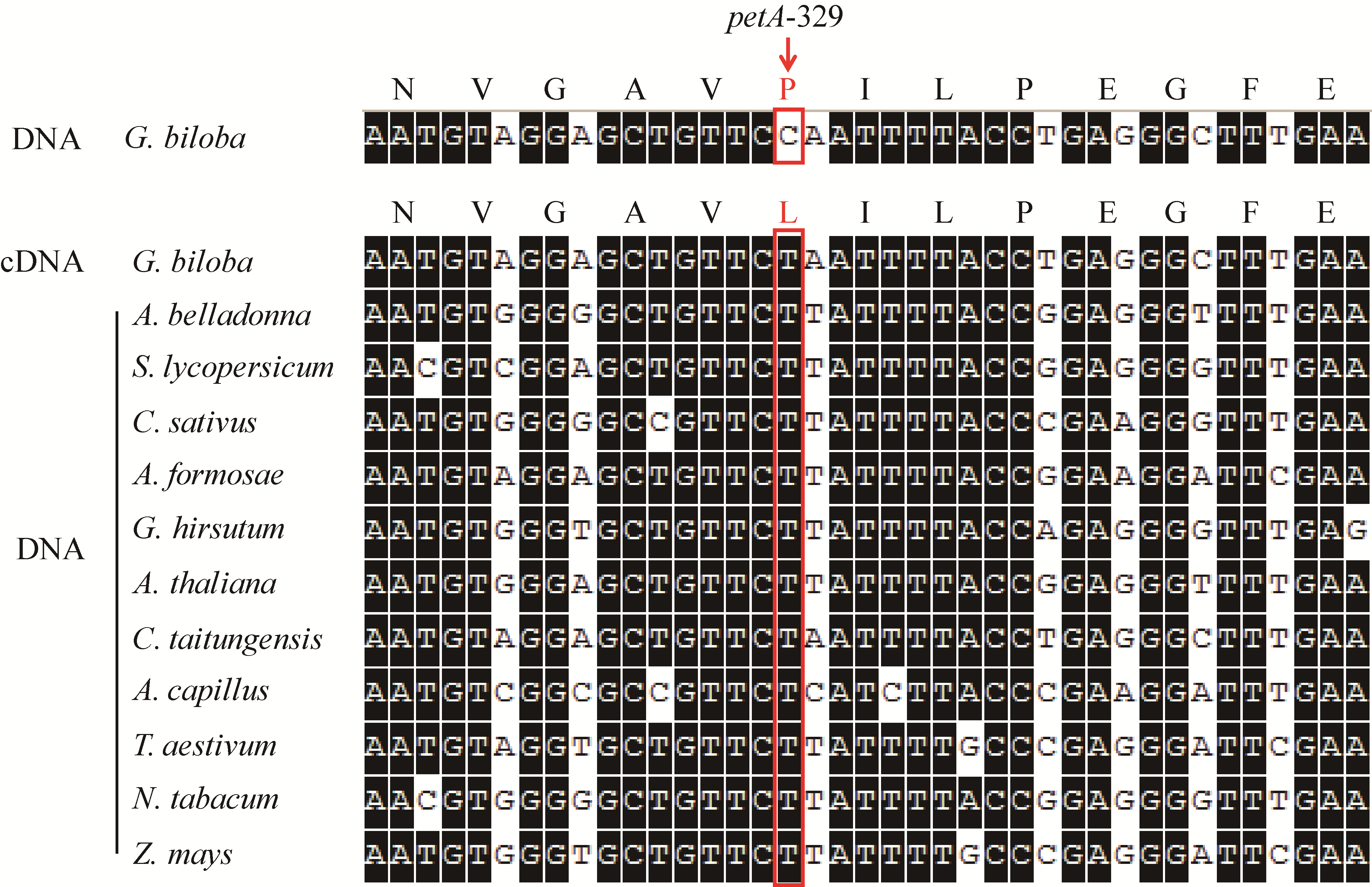


**Additional Figure 4. Multiple sequence alignment of *psaA* in 12 species.**

The red arrow indicates RNA editng site at *petA*-329.
